# Foundation artificial intelligence models enable high-accuracy diagnostic differentiation of hybrid neurofibroma/schwannoma using whole-slide images

**DOI:** 10.1016/j.jpi.2026.100700

**Published:** 2026-07-28

**Authors:** Fabio Hellmann, Maxim Anokhin, Pascal Schimmler, Daniel Tippner, Stefan Plontke, Catena Kresbach, Martin Mensah, Elisabeth André, Anja Harder

**Affiliations:** a Human-Centered Artificial Intelligence, University of Augsburg, Augsburg, Germany; b Medical Centre for Neurosurgery/Neuroradiology, Gensingen, Germany; c CURE-NF Research Group, Medical Faculty, Martin Luther University Halle-Wittenberg, Halle (Saale), Germany; d Department of Otorhinolaryngology, Head and Neck Surgery, Martin Luther University Halle-Wittenberg, Halle (Saale), Germany; e Institute of Neuropathology, University Hospital Hamburg-Eppendorf, Hamburg, Germany; f Department of Human Genetics, Helios Klinikum Berlin-Buch, Berlin, Germany; g Institute of Clinical Research, MSB – Medical School Berlin, Berlin, Germany; h Institute of Neuropathology, University Hospital Münster, Münster, Germany; i Department of Life Sciences, Reutlingen University, Reutlingen, Germany

**Keywords:** Schwannoma, Neurofibroma, Hybrid, Nerve sheath tumor, Whole slide

## Abstract

Peripheral nerve sheath tumors encompass a heterogeneous group including schwannoma, neurofibroma, and hybrid neurofibroma/schwannoma (HNS). Accurate differentiation is crucial due to distinct biological behavior, different management, and a possible assignment to a genetic disease. We investigated whether deep learning (DL)-based artificial intelligence (AI) reliably distinguished HNS from schwannomas and neurofibromas using whole-slide images (WSIs). Utilizing a dataset of H&E-stained WSIs from 115 tumors, we applied state-of-the-art foundation models (UNI, CONCH, ResNet50) for feature extraction, combined with multiple instance learning algorithms CLAM and mMIL, and a patch-based classifier.

Our models achieved high validation performance, with macro-area under the receiver operating characteristic curve (AUC-ROC) values up to 1.0 for UNI-based MIL models. Attention maps correlated well with annotations. HNS were reliably distinguished from schwannoma and neurofibroma using a pretrained AI workflow that was easy to interpret. The choice of foundation model and stain normalization emerged as major performance drivers, with UNI-based mCLAM achieving the highest validation performance, whereas a ResNet50-based mCLAM model with stain normalization reached the best HNS accuracy (0.96) and F1 (0.98) on the test set. Patch-level classifiers with CONCH and UNI embeddings showed slightly lower three-class performance on the validation set (macro AUC-ROC approximately 0.8–0.87), but supported data-driven thresholds for the proportion of Sw-like and Nf-like tissue used for slide-level HNS classification, and a ResNet50-based patch model achieved an HNS accuracy of 0.79 and F1 of 0.88 on the test set.

We demonstrated that minimal preprocessing, combined with an interpretable output, quantified tumor components with very high accuracy. This study supports the feasibility of DL tools for the nuanced differentiation of schwannoma and neurofibroma from HNS. Our approach can therefore improve diagnostic accuracy. Limitations include the dataset, technical artifacts, and discrepancies between validation and test performance across encoders. Therefore, future work should include external validation across scanners, labs and centers, protocols, prospective assessment of workflow integration, and benchmarking against interobserver variability to confirm clinical utility and generalizability.

## Highlights

• First deep learning framework for automated diagnosis of hybrid nerve sheath tumors using deep learning on H&E slides.
• Foundation models enable a robust differentiation of tumor types, and interpretable heatmaps localize distinct regions.
• AI-derived thresholds allow objective identification of hybrid neurofibroma/schwannoma correlating withannotations.

## Introduction

### Background

Peripheral nerve sheath tumors (PNST) are a heterogeneous group of neoplasms originating from structures of peripheral nerves and encompass both benign and malignant entities.^1^, ^2^, ^3^ PNST can occur sporadically or as part of tumor syndromes in various parts of the body, in typical as well as rare locations.^4^, ^5^ The 5th WHO edition of Soft Tissue and Bone Tumors lists schwannoma (Sw), neurofibroma (Nf), perineurioma, hybrid nerve sheath tumor, and others within the subchapter of PNST. In contrast to the 5th WHO edition of the Central Nervous System Tumors, the soft tissue classification does not recognize a WHO grade for PNST, but a similar ICD classification system.^4^, ^5^ PNST originate from Schwann cell-derived cells, although the cellular composition includes other components.^6^

### Rationale

**Fig. 1:**
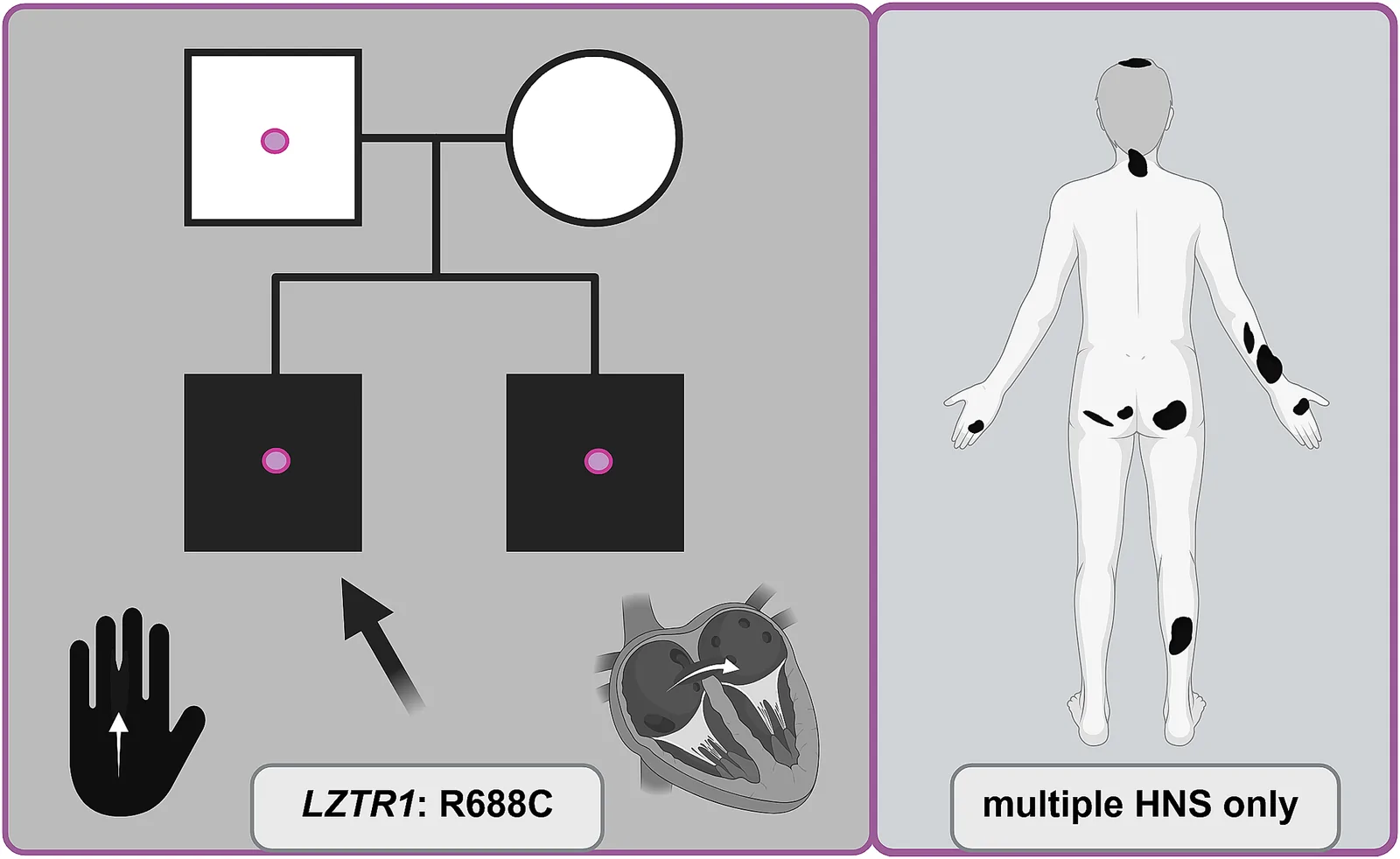
Multiple hybrid neurofibromas/schwannomas (HNS) in LZTR1-SWN. Pedigree of a family presenting with multiple HNS only (LZTR1-SWN was diagnosed in two sons carrying the pathogenetic *LZTR1* germline variant c.2062C > T/p.Arg688Cys in exon 17 (colored dots). The index patient demonstrated on the left side (arrow) has been previously described as patient 9^9^; at that time, his *LZTR1* mutation was not yet proven. The two brothers presented a similar phenotype of exclusively and histologically characteristic HNS. These tumors showed monosomy 22 and loss of *LZTR1* expression. Besides multiple locations of HNS, clinical data included syndactyly III/IV (index patient I) and a heart defect (foramen ovale defect) in patient II. There were no facial nor minor/mosaic signs of NF2-SWN. A mild heart defect and syndactyly might point to minor Noonan-like features (for a review of cardiac features in *LZTR1* mutants, see Busley et al.^10^). Both parents did not display any abnormalities. Created in BioRender. Harder, A. (2026) https://BioRender.com/uphsuzm (RO29ISGCM2).

Accurate distinction between Sw, Nf, and hybrid PNST (HPNST) is clinically relevant because it may support the identification of an underlying tumor predisposition. Whereas benign tumors like Sw and Nf are common, malignant PNST (MPNST) pose a significant clinical challenge due to their poor response to conventional therapies.^7^ HPNST have increasingly been recognized and illustrate the complexity of PNST with mixed histological features and their association with genetic disorders such as neurofibromatosis type 1 (NF1) and schwannomatosis (SWN). There is a strong correlation between the occurrence of Sw in SWN, Nf in NF1, and hybrid neurofibromas/schwannomas (HNS) in various SWN, as exemplified by Leucine-zipper-like transcriptional regulator 1 (LZTR1)-SWN in Fig. 1.^8^

**Fig. 2:**
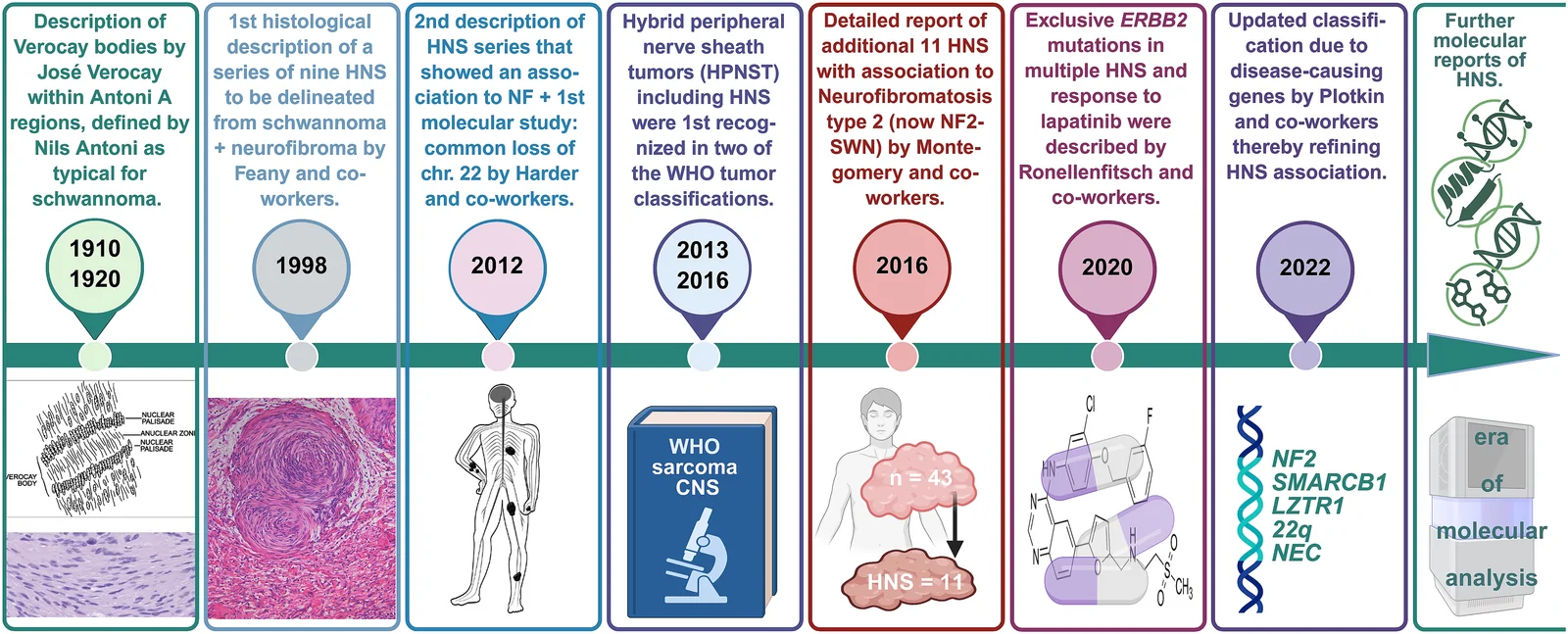
History of the description and reception of hybrid neurofibroma/schwannoma (HNS). Series of HNS have been reported in only a few studies so far. It took 80 years to differentiate them from conventional schwannoma in 1998^13^ and more than a decade to demonstrate a clear association to neurofibromatosis, especially to Schwannomatosis (SWN).^12^, ^14^ Thereafter HNS was integrated into tumor WHO classifications and upcoming molecular techniques allowed a more comprehensive routine mutation screening in benign tumors leading to detection of additional mutations such as in *ERBB2, KMT2,* and others.^1^, ^2^, ^9^, ^15^, ^16^, ^17^ Created in BioRender. Harder, A. (2026) https://BioRender.com/zf8omc6 (FG29QGZHIV).

In 2022, HNS was included into the updated diagnostic criteria of SWN, which is now divided into subtypes (NF2-, SMARCB1-, LZTR1-, 22q-related- SWN and SWN-NOS and -NEC) according to the gene involved (Fig. 2).^11^ Looking back, one must assume, for the sake of correction, that HNS seems to occur primarily in SWN. Occurrence in NF1 may have been misjudged due to imprecise or superficial clinical characterization. In our experience, HNS can occur both alongside Sw and exclusively at multiple localizations simultaneously. HNS can also exhibit a plexiform growth pattern, leading to misdiagnosis of a plexiform Nf. The first larger case series of the to date underrecognized entity of HNS was published in 2012, although 11 reports, including a larger study by Feany et al.,^12^, ^13^ had been published before (Fig. 2).

**Fig. 3:**
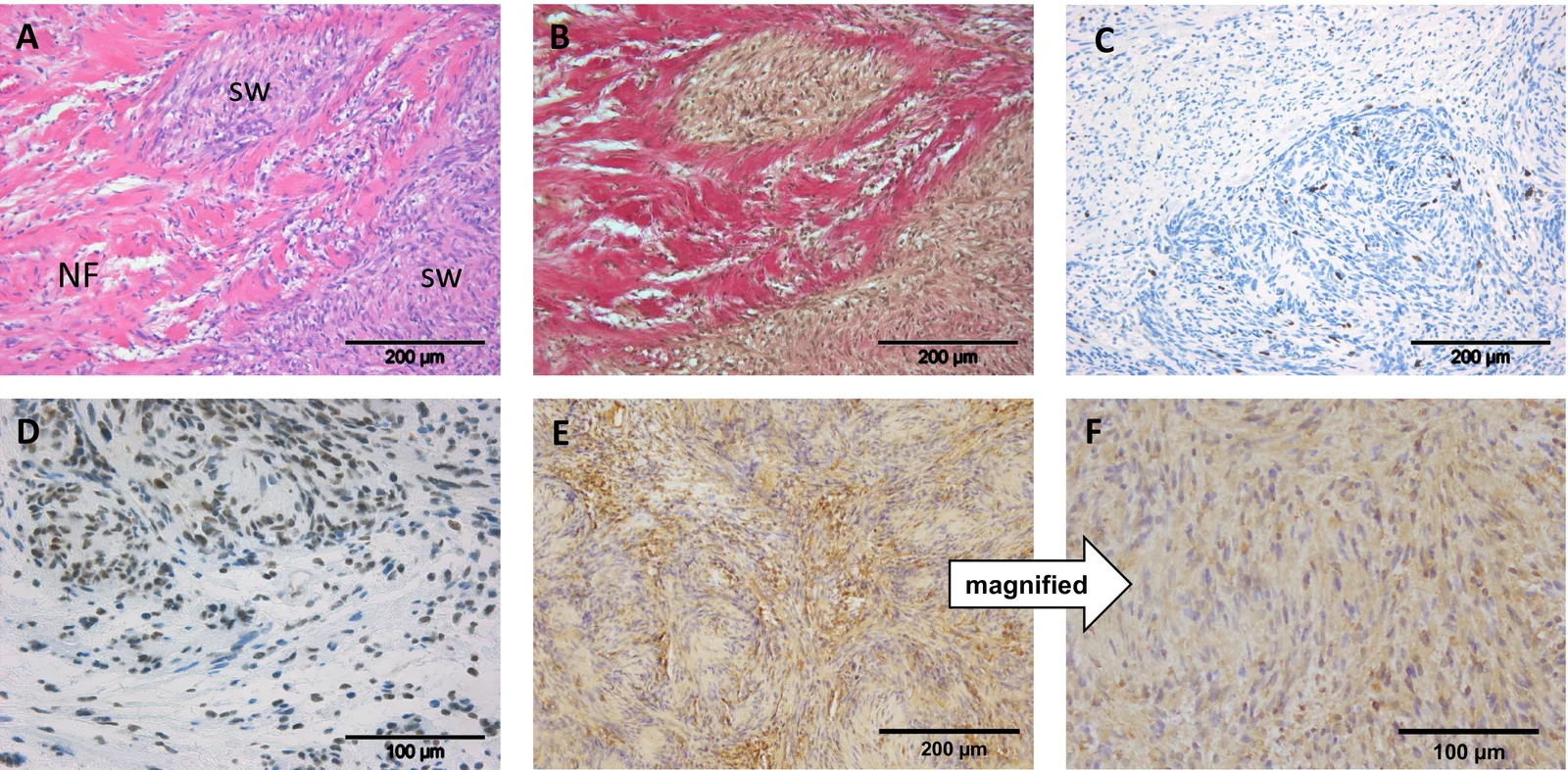
Typical histopathology of hybrid neurofibroma/schwannoma (HNS). Features of a classical HNS in a patient with LZTR1-SWN (see Fig. 1). Representative histological pattern of one tumor (right forearm). The upper panel (A—H&E, B—Elastica van Gieson stain, C-Ki67 immunohistochemistry) demonstrates two distinct areas of SW-like (SW) and NF-like (NF) growth patterns within the same tumor. The NF area is rich in collagen (B). Proliferation (Ki67 index) is low (<5%). The lower panel shows immunohistochemistry for SMARCB1 (D: intermingled positive and negative nuclei; percentage of immunopositive nuclei is >50%) and for LZTR1 (E and F). LZTR1 is lost in many but not all tumor cell nuclei, with a weak cytoplasmic staining visible focally.

Only a few studies have attempted molecular characterization to date^9^, ^14^, ^15^, ^18^: those uncovered monosomy of chromosome 22 or loss of 22q,^9^ pathogenic variants of *LZTR1*, *NF2*, *SMARCB1* on chromosome 22, *CTNNA3, ERBB2, RET*, *KMT2A*, and the gene fusion *RREB1::LPP*.^9^, ^15^, ^16^, ^17^, ^18^ Integrating molecular characterization, HNS was most commonly reported across different SWN subtypes and less frequently in sporadic cases.^8^ Recently, APOD was suggested as a discriminator between NF-like and Sw-like areas in HNS.^19^ In general, molecular data on HNS are sparse. HNS was included in the 4th WHO edition of the Classification of Tumors of Soft Tissue and Bone in 2013^20^ and only later in the 4th WHO edition on the CNS in 2016^21^ (Fig. 2), which explains why it was probably not well received until then and was diagnosed either as Nf or Sw to best fit the categorization at the time. To date, this tumor entity appears to be underdiagnosed, which continues to complicate the collection of cases. Typical histological features of HNS (Fig. 3, Material and Methods) include both areas typical of a Nf corresponding to a Nf grade I (CNS WHO classification) and of areas typical of a Sw fulfilling at least the criteria for Antoni A regions of a Sw grade I (CNS WHO classification). Furthermore, sparse case series highlight the need for a standardized approach to reliably differentiate HNS from benign Nf and Sw, enabling better attribution to an associated tumor syndrome, which is important for the correct clinical work-up and therapy.^8^ Although it should not be particularly difficult to distinguish histologically between areas typical of Sw and Nf, some cases still pose a challenge (Fig. 3). Especially, if the Sw-like area is small or if there are major regressive changes, one might overlook this entity.

### Objective

The objective of this study was to develop and evaluate a system for the automated histological and differential diagnosis. We aimed to develop a robust, simple method based on artificial intelligence (AI) to support the diagnosis of HNS in difficult cases and to provide reliable determination to rule out the differential diagnosis of HNS when certainty is desired. An AI-based approach for a benign lesion could also be applied instead of molecular analysis and could assist pathologists.

Computer-aided diagnosis (CAD) systems powered by deep learning (DL) are reliable assistants in processing large datasets or providing second opinions as shown for image analyses, especially in tumor segmentation and classification.^22^, ^23^, ^24^ Thus, over 90% accuracy was achieved in distinguishing glioblastomas from lower-grade gliomas.^25^ In this study, the ability of DL algorithms to autonomously extract hierarchical features from histopathological images makes them highly generalizable also in PNST. As application to automated PNST diagnosis remains underexplored, the specific objective was to start with a focus on HNS being a true intermediate entity from a pathologist's perspective. We hypothesize that an AI-based model can accurately differentiate between the three entities: SW, Nf, and HNS.

## Materials and methods

### Selection and diagnostic confirmation

All formalin-fixed, paraffin-embedded (FFPE), completely anonymized samples initially underwent pathological investigation by at least two pathologists/neuropathologists before case selection.

The final diagnosis for inclusion into the study was done according to the 5th edition of the WHO Classification of Tumors of Soft Tissue and Bone and Central Nervous System Tumors.^1^, ^2^ Given the limited scope of essential criteria in the WHO classification, we propose a set of histopathological criteria for HNS to assist with routine diagnostic evaluation (Table 1). Thus, we defined five major criteria that all needed to be fulfilled to include the HNS in the study. These diagnostic criteria include all the features we consider important.

**Table 1:** Diagnostic criteria to separate hybrid neurofibroma/schwannoma (HNS) from schwannoma (Sw) and neurofibroma (Nf).

*Major* criteria—gross architecture	1. Both tumor parts must occur within the same lesion (external Nf-like areas are typical, Sw-like areas are often located more within the tumor), simple collision tumors are excluded.
	2. The areas typical of a Nf meet the criteria for a Nf (grade I CNS WHO classification) completely (pronounced collagen fiber formation is typical).
	3. The areas typical of a Sw completely fulfil at least the criteria for Antoni A regions of a Sw (grade I CNS WHO classification).
*Major* criteria—differential diagnosis	4. At least 15% or (when less very clear, diagnostically unambiguous) areas of one and the other tumor areas should be present, so that both areas can be reliably diagnosed as Nf and Sw (smaller areas might cause problems for allocation and should be diagnosed very carefully).
	5. It must be ensured that the areas typical of Nf are not Antoni B areas (Nf areas contain collagen fibers and a mixture of Schwann cells and fibroblasts).
*Minor* criteria	6. The tumors can also occur as plexiform tumors. Here, you can also identify abundant perineural cells within and among the capsule structures.
	7. Pronounced lymphocytic infiltrates are frequent in the periphery of the lesion.
	8. Demarcation of the Nf and Sw components is usually very sharp.

### Dataset

Tumor specimens were provided from the archives of the Institute of Neuropathology at Muenster (University Hospital Münster), the Institute of Neuropathology Hamburg (University Hospital Hamburg), and the Institutes of Pathology and Neuropathology Mainz (University Medicine Mainz) in Germany. In total, 115 tumors (375 histological slide sections, H&E) were analyzed, comprising 36 Nf, 50 Sw, and 29 HNS. The specimens were digitized using two different scanning systems: Nano Zoomer 2.0HT scanner (HAMAMATSU PHOTONICS K. K., Hamamatsu City, Japan) and Axio Scan.Z1 slide scanner (ZEISS, Carl-Zeiss-Promenade 10, 07745 Jena, Germany). All slides were scanned at a 40× magnification for high-resolution imaging and to facilitate detailed examination of all histological structures. The scanned specimens were identified using NDPview 2 and Zeiss ZEN 3.11 (Carl Zeiss Microscopy GmbH) software. Selected tumor areas of Nf and Sw were segmented (delineated, annotated, and extracted) using NDP.toolkit 2.3 (Hamamatsu Photonics K.K., Hamamatsu City, Japan) and of HNS using QuPath (an open-source software for bioimage analysis).^26^

### Region of interest

After quality assessment of digitalization, we manually marked the regions of interest (ROIs) by two different approaches for the training set. Using the NDP.toolkit 2.3 (Hamamatsu Photonics K.K., Hamamatsu City, Japan) and the open-source software for bioimage analysis QuPath (available at https://qupath.github.io) areas were traced around with a virtual pen to define the typical areas of Sw and Nf and thereby selected to be cut for further processing (Supp. Fig. 1). We also excluded artifacts, atypical tissue components, or unclear histology by these approaches. The goal was to obtain a higher-quality sample of the tumor type for the subsequent training of different models. The HNS slides were annotated with the regions of Sw and Nf to visually and qualitatively compare to the models' predictions The identification of typical tumor areas was always defined by two observers including an experienced neuropathologist (AH) and according to the above defined criteria. Manual ROI annotations were used exclusively for training data curation and were not involved in model tuning, threshold selection, or performance evaluation. For validation, WSIs were analyzed without any manual intervention. ROI annotations were generated retrospectively for the validation set solely to enable qualitative comparison between annotated regions and the model's predictions (Supp. Fig. 2), without influencing the evaluation metrics.

### Image preprocessing and feature extraction

**Fig. 4:**
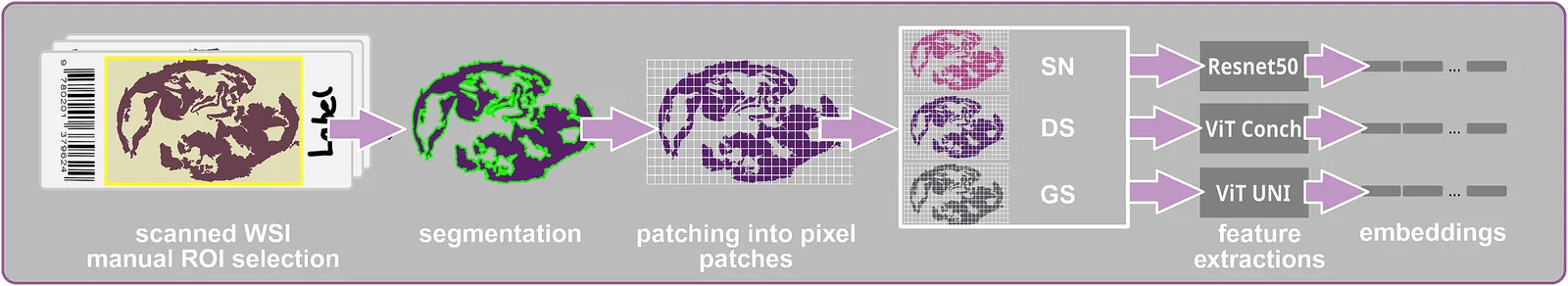
Preprocessing pipeline. The pipeline processed the raw input of a WSI scan with its barcode and handwritten label in several steps by manual ROI selection of the tissue area, separating the tissue from the background, slicing the tissue into 224 × 224-pixel sized patches, normalized them in three different ways (stain normalization (SN), default/unaltered (DS), and grayscale (GD)) and utilized three pretrained models (ResNet50, CONCH, and UNI) to extract features from those patches. Created in BioRender. Harder, A. (2026) https://BioRender.com/a8m6r49 (JP29ISH5DW).

As scan preprocessing is a vital step before a classifier is trained on the data, we have developed a preprocessing pipeline consisting of five steps, which were applied before our models' training: WSI scanning, manual ROI selection, segmentation, patching, normalization, and feature extraction (Fig. 4).

To remove the barcode and handwriting from the scanned WSI for further processing, the ROI of the HNS containing the tissue to be analyzed was manually cropped. To separate tissue regions and remove the background from the image, OpenCV's function to find contours with the “RETR_CCOMP” retrieval mode and “CHAIN_APPROX_NONE” approximation method was applied for tissue segmentation onto the cropped digitized glass slides. Placing the segmented tissue in an image, the surrounding area pixels were set to zero (dark area with green borders in Fig. 4). Afterward, tissue-containing areas were partitioned into standardized 224 × 224-pixel-sized patches at a magnification level of 20 to enable systematic analysis. Peripherally, the patches contained not only tumor tissue but also surrounding non-tumor regions. After converting the WSI into patches, the pipeline was split into three branches to evaluate the impact of stain variations on the models' training. This resulted in three image alterations: default (unaltered) patches, grayscale patches, and stain-normalized patches. Stain normalization was performed with the Macenko algorithm,^27^ using a target stain vector derived from a reference image. This normalization led to a distinct separation between eosin and hematoxylin stain vectors, as confirmed by principal component analysis. Finally (step 5), patches were converted into numerical representations, so-called feature embeddings. We used computer vision models, which have been trained on large pathology datasets and are suitable for downstream classification tasks. These models used three pretrained foundation models: a general-purpose self-supervised model for pathology (UNI) with pretrained weights from Chen et al.,^28^ CONtrastive learning from Captions for Histopathology (CONCH) with pretrained weights from Lu et al.,^29^ and a 50-layer Residual Network (ResNet50).^30^

### Overview of the models used

The classification of the given problem was tackled from two different angles. On the one hand, three multiple instance learning (MIL) algorithms (Supp. Fig. 3) were implemented to classify at the WSI level. On the other hand, a patch-based classifier was used to classify the patches into which the WSI was sliced.

The multiclass MIL (mMIL),^31^ a baseline comparison method (consisting of two sequential fully connected layers with ReLU activation functions), was used as a straightforward approach, though it is likely limited by its simplicity in handling complex histopathological patterns. It used max pooling for classification. The chosen (preselected) hyperparameters for mMIL were as follows: 10 epochs of training with a patience of 10, a learning rate of 0.0002, weight decay of 0.00001, cross-entropy bag loss, dropout of 0.1, early stopping, 5-fold cross-validation (CV), and the Lookahead (RAdam).

### Clustering-constrained attention multiple instance learning

We implemented the Clustering-constrained Attention Multiple instance learning (CLAM)^32^ and created a modified CLAM (mCLAM) to address the fact that Sw and Nf are considered mutually exclusive despite sharing some features (e.g., blood vessels), whereas HNS contain elements of both. The mCLAM used attention scores only from the two primary classes (Nf and Sw) to train instance classifiers. Usually, CLAM modifies the embedding space during training by ranking embeddings using attention scores (for each class, the highest and lowest ranked embeddings are used to define positive and negative classes for clustering). The mCLAM omitted the hybrid branch from the instance classifier training, thus creating a specialized bag classifier for HNS, and incorporating information from both primary branches. The aim was to reduce the impact of HNS-specific artifacts that are not reinforced by instance classifiers. The selected hyperparameters for CLAM and mCLAM were as follows: 200 epochs of training, patience of 20, learning rate of 0.0002, weight decay of 0.00001, cross-entropy bag loss, dropout of 0.25, early stopping, 5-fold CV, and the Adam optimizer. The bag weight was set to 0.7, and the instance loss function was a support vector machine (SVM)-based one. The number of top and bottom instances used for clustering was 8. The model type was clam_mb (multibranch), and the model size was small.

### Patch-level classifier

This classifier consisted of three fully connected layers with dropout for regularization and employed weighted random sampling to address potential imbalances between classes Nf and Sw (Supp Fig. 4). For whole-slide classification, the system used custom pooling based on dataset-derived thresholds, calculating the percentage of area classified as each class for training WSIs and using these thresholds to classify HNS cases in the test set. We applied this method because it assumes a high witness rate (most patches share the label of the slide they are contained in), making it interpretable and artifact-resistant compared to attention-based approaches. Additionally, it allowed for exploring the thresholds at which a given sample is, in fact, an HNS. The hyperparameter grid search helped identify the best configuration for training the classifier, using solely stain normalization, different input sizes (512 and 1024), batch sizes (32, 64, 128), dropout rates (0.2, 0.3, 0.5), and hidden layer sizes [32/64/128, 16/32/64]. The static hyperparameters were learning rate (0.001), number of epochs (1000), number of outputs (1), patience (200), early stopping, and 5-fold CV.

### Evaluation methods

**Fig. 5:**
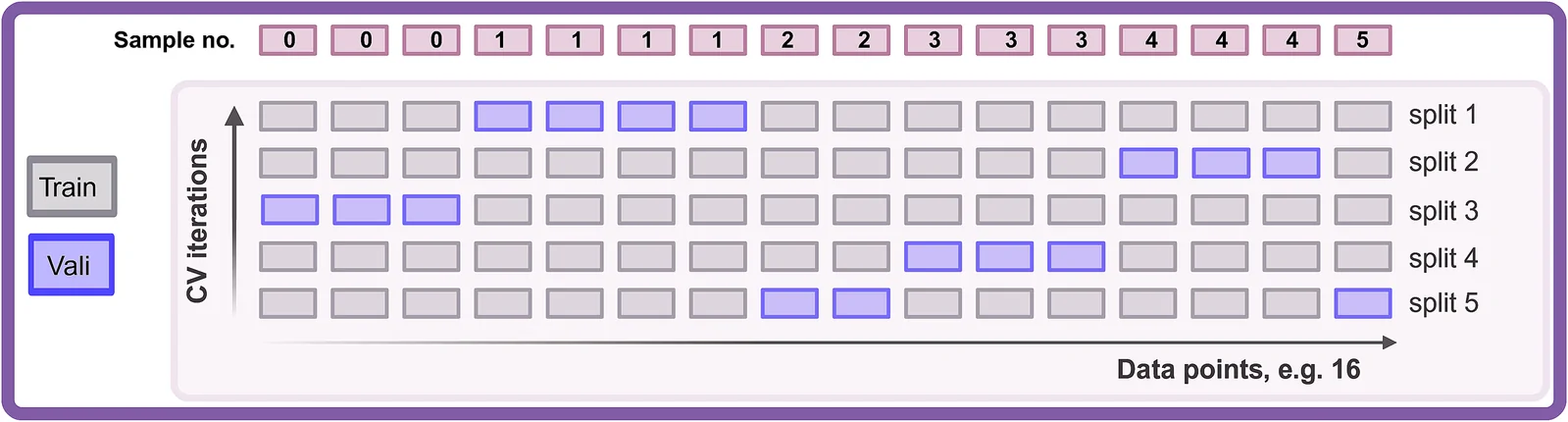
Dataset description. The illustration provides an exemplary overview of how the sets were divided, because the actual numbers would be too extensive. Created in BioRender. Harder, A. (2026) https://BioRender.com/jykttev (NA29ISHI2G).

To ensure correct validation of our diagnostic model, we employed a 5-fold CV with dual stratification—maintaining balanced representation across both individual patients and diagnostic classes set (see Fig. 5). This stratification approach prevents case clustering bias that could occur if multiple tissue sections from the same patient were inadvertently split between training and validation sets. We defined the 17 HNS slides from two scanners as a hold-out test set to verify the generalizability of our models to other scanners/centers. A 5-fold CV divided the dataset from another center into five equal parts (folds). Training the model in four folds, testing on the remaining fold, and repeating the process five times ensured the model's robustness. This dataset, split into training, validation, and test sets, was kept constant across all experiments.

The models' performance was assessed using standard diagnostic accuracy metrics: the area under the receiver operating characteristic curve (AUC-ROC), which measures discriminatory power, overall diagnostic accuracy per class, and balanced accuracy throughout all classes. To measure the AUC-ROC, a plot of the true-positive rate (sensitivity) against the false-positive rate (1—specificity) is created at various threshold settings to create the ROC curve. The AUC is computed as the AUC-ROC curve. An AUC-ROC approaching 1.0 indicates excellent diagnostic discrimination between positive and negative classes (=100% sensitivity and 100% specificity), whereas values around 0.5 suggest no superiority over random classification. Additionally, we present Precision, Recall, and F1-scores for more detailed performance information.

Because the patch-level classifier supports only two classes, Nf and Sw, we established two thresholds, lower (c1) and upper (c2), to delineate diagnostic categories based on the proportion of schwannoma-positive regions within each WSI. To ensure accurate threshold setting, the best model configuration, optimized by MCC, was evaluated on the training set for each fold. For each model and HNS samples, thresholds a and b are computed using a three-class adaptation of Youden's J statistic^33^ proposed by Nakas et al.^34^ This method is mathematically formalized for ordered classes (with Nf ≤ HNS ≤ Sw), and seeks threshold values that maximize the total Youden index for the classification boundaries:

$$J={\left(\frac{\mathrm{TP}}{\mathrm{TP}+\mathrm{FN}}+\frac{\mathrm{TN}}{\mathrm{TN}+\mathrm{FP}}-1\right)}_{\left(\mathrm{Nf},\mathrm{HNS}\right)}+{\left(\frac{\mathrm{TP}}{\mathrm{TP}+\mathrm{FN}}+\frac{\mathrm{TN}}{\mathrm{TN}+\mathrm{FP}}-1\right)}_{\left(\mathrm{HNS},\mathrm{Sw}\right)}.$$

This approach investigated 5050 threshold combinations per fold to optimize discriminatory performance. For patch classification, the threshold was selected from 1000 evenly distributed values spanning the classifier output range (0–1 or 0%–100% probability of Sw). For each threshold, both sensitivity and specificity were calculated, and the threshold that maximized the standard Youden's J statistic^33^ was chosen as the optimal decision boundary.

To evaluate whether the computational model identified clinically relevant morphological features that align with established pathological criteria and expert diagnostic reasoning, and to ensure the AI's decision-making process aligns with histopathological interpretation, we conducted qualitative validation by comparing the XAI-generated visualizations against expert pathologist annotations on the same tissue sections.

## Results

### Differentiation through various classifiers of the applications

**Fig. 6:**
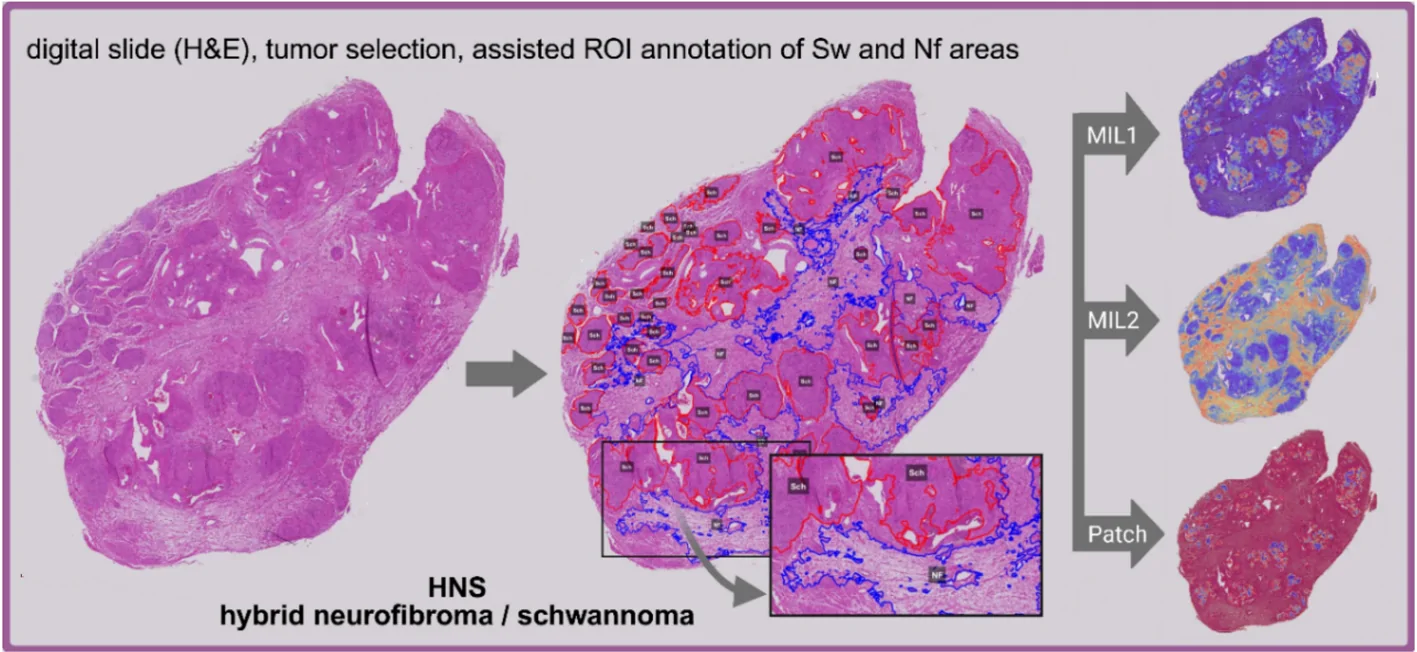
Results of the classifiers with the highest AUC-ROC. Visualizations of WSI classifications using the mCLAM and patch-level classifier. Both MIL visualizations, produced by our mCLAM approach, show attention scores via color coding, where red indicates high attention and blue indicates low attention—with MIL1 focusing on classifying Sw and MIL2 focusing on classifying Nf. The patch-level classifier uses a color scale from red (Nf) to blue (Sw), where each patch is classified independently and then placed back into the WSI with its corresponding prediction. The patches overlap by 50% to produce a more detailed heatmap. Created in BioRender. Harder, A. (2026) https://BioRender.com/9o9yy44 (BY29ISI03Y).

We tested three different DL approaches on the validation split per fold and computed the mean across all folds, along with their standard deviations and confidence intervals. Two MIL-based models and one patch classifier, with various configurations, were used to classify Nf, Sw, and HNS tumors based on H&E stainings (Fig. 6). Among the MIL, CLAM, and mCLAM algorithms, the models showed different results. The following paragraphs will show the results of all trained classifiers in depth.

### Validation set benchmark of MIL, CLAM, and mCLAM algorithms

The algorithms were analyzed using three normalization techniques and three encoders, as described. Results for the UNI, CONCH, and ResNet50 encoder types are shown in Supplementary Table 1.

The mCLAM with the UNI encoder and stain normalization achieved the highest performance in our ablation study, with an MCC of 0.95 ± 0.05 and a confidence interval (CI) of 0.94–1.00 (see the confusion matrices for each CV fold in the Supplementary Fig. 7). The MIL algorithms, except for the UNI encoder and default normalization, achieved the lowest validation set results. The mMIL with the UNI encoder and default normalization performed slightly better, but is not among the top 10 performing models.

### Validation set benchmark of patch-level classifier

The patch-level classifier ablation study (Supp. Table 2) for finding optimal hyperparameters using grid search across several combinations of hidden layer sizes, dropout rates, batch sizes, normalization techniques, and encoder models. The Youden J Statistics was applied to all patch-level classifiers to compute the c1 and c2 thresholds before evaluating their performance on the validation set. The best-performing classifiers used either the CONCH or the UNI encoder, and all used the stain normalization method. The best-performing patch-level classifier in the ablation study also used an UNI encoder and stain normalization, with hidden sizes of 64 and 32, dropout of 0.5, and a batch size of 128, and achieved, based on the c1 and c2 thresholds, the highest MCC with the lowest standard deviation of 0.77 ± 0.13.

**Fig. 7:**
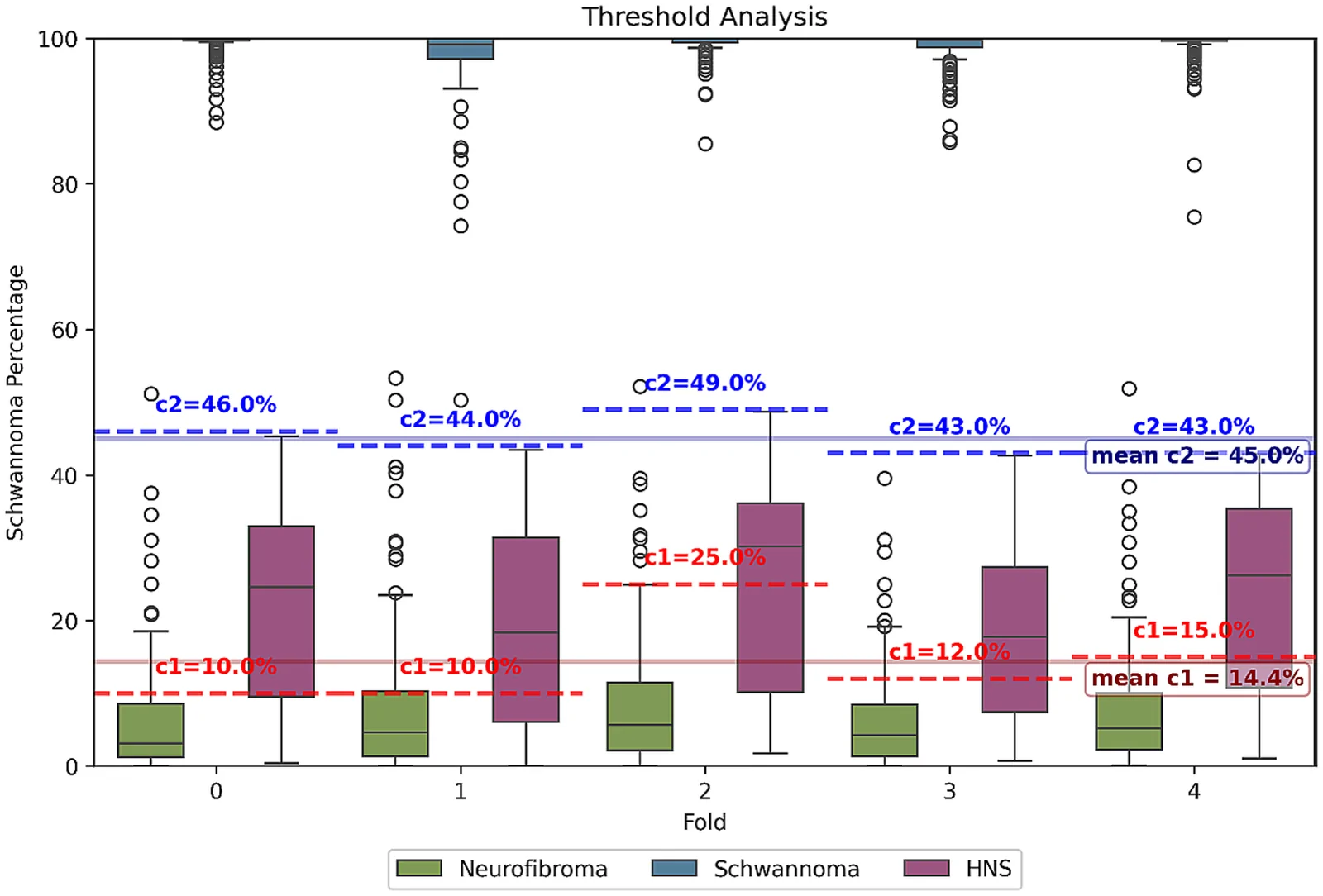
Boxplots describing the distribution of the percentage of schwannoma within each WSI of the training set for each fold and all folds combined. Each box plot illustrates the spread of all classes. The red dashed line represents the lower threshold (*c1*) for separating neurofibroma from HNS tissues. The blue dashed line represents the upper threshold (*c2*) for distinguishing HNS from schwannoma.

The distribution of Sw percentage for each tissue class (Sw, Nf, and HNS) is visualized in Fig. 7 across five CV folds. The training data within each fold was used to compute the optimal c1 and c2 thresholds, which were then evaluated on the validation set displayed in Fig. 7. Each box plot illustrates the spread and central tendency for HNS. The lower threshold (*c1*) for separating Nf from HNS tissues is at a mean Sw area of 14.4% (red dashed line). The upper threshold (*c2*) for distinguishing HNS from Sw is at a mean of 45.0% Sw area (blue dashed line).

### Test set benchmark

To test the models' generalizability to other scanners and centers, we held out data from another center/scanner. Based on this test set, we benchmarked the models' performance on the HNS cases only. For the patch classifier, the Youden's J statistic was used, and the computed thresholds (c1, c2) were applied to classify the slides.

The results in supplementary table 3 show that the best-performing model is the MIL-based mCLAM with a ResNet50 encoder and stain normalization, achieving HNS accuracy of 0.96 ± 0.05 and F1 of 0.98 ± 0.03. The second-best model is the patch-level classifier, which also uses the ResNet50 encoder and stain normalization, with c1 = 0.07 ± 0.02 and c2 = 0.44 ± 0.04, achieving a HNS accuracy of 0.79 ± 0.03 and an F1 of 0.88 ± 0.02.

## Discussion

### Proof-of-principle

**Fig. 8:**
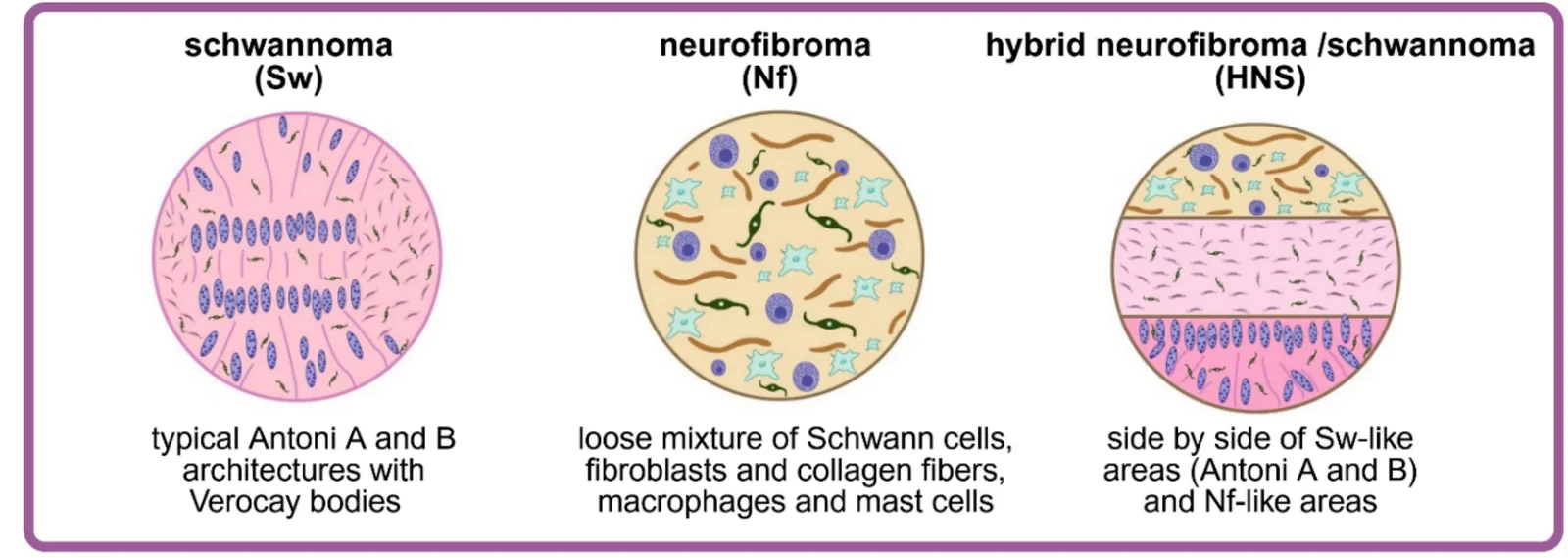
Schematic representation of the characteristics of schwannoma (Sw), neurofibroma (Nf), and hybrid neurofibroma/schwannoma (HNS). Created in BioRender. Harder, A. (2026) https://BioRender.com/p4wnrhd (CR29ISJ571).

We present one of the first histology-based AI approaches for the PNST entity HNS, demonstrating robust separation from conventional Nf and Sw using a compact combination of foundation encoders, MIL methods, and a lightweight patch-based model. Prior work in this domain has focused on radiomics or combined clinical–imaging models.^35^, ^36^ In contrast, our results show that H&E-based DL alone achieves high differential diagnostic performance for HNS, Nf, and Sw (Fig. 8), although with sensitivity to encoder choice and training paradigm. In real-world workflows, these models are best understood as task-specific decision-support tools that distinguish HNS, Nf, and Sw, act as a “second reader” to highlight suspicious hybrid regions and quantify relative Nf and Sw components, rather than as general-purpose PNST classifiers. Beyond diagnostic support, reliable identification of HNS may facilitate investigation of its biological underpinnings. A key open question is whether the Nf- and Sw-like components share a common molecular origin or reflect distinct or sequential genetic events. It has been proposed that Nf-like areas may arise as a morphological variant associated with increased Ras/MAPK signaling, potentially requiring additional alterations relative to the Sw-like area.^9^, ^18^ Accurate distinction from classical Nf and Sw is therefore essential, including in the context of SWN.

### Model performance and encoder effects

In slide-level evaluation, UNI-based mCLAM and CLAM models achieved excellent performance with macro-AUROC of 1.00, micro-F1 of 0.96–0.97, and balanced accuracy of 0.94–0.97 for three-class discrimination (Sw, Nf, and HNS). Attention maps correlated well with pathologist annotations, highlighting diagnostically relevant HNS-like regions rather than technical artifacts. The test set benchmark confirmed cross-center generalizability of MIL-based HNS detection: an mCLAM model with a ResNet50 encoder and stain normalization achieved HNS accuracy of 0.96, F1 of 0.98, and perfect recall, supporting its suitability for sensitive triage. Notably, the top-performing test models relied on ResNet50 despite inferior validation performance, indicating dataset-dependent encoder behavior.

Patch-level classifiers provided complementary spatial granularity and enabled quantitative thresholding of tissue composition. Using CONCH and UNI embeddings, internal macro-AUROC ranged from 0.8 to 0.87, with balanced accuracy of 0.75–0.81, consistently below MIL performance. On the test set, the best patch model (ResNet50 with stain normalization) achieved HNS F1 of 0.88 and accuracy of 0.79, demonstrating robustness under distribution shift. However, the same validation-test discrepancy persisted, with ResNet50 outperforming on test data but underperforming during validation, underscoring sensitivity to encoder choice, data origins, and scanning variability.

Aggregation of patch predictions into tissue proportions enabled derivation of data-driven HNS thresholds. The best validation model classified HNS at Sw-like coverage of 14.4%–45.0% and Nf-like coverage of 55.0%–85.8%, but generalized poorly (test HNS accuracy ∼0.39, F1 ∼0.55; rank 81/135; c1 ∼14.4%, c2 ∼45%), indicating near-random performance. In contrast, the best test-set patch model (rank 2/135; accuracy ∼0.79, F1 ∼0.88) ranked only 38/108 on validation, with shifted thresholds (c1 ∼7%, c2 ∼46%). Practically, the HNS-Sw boundary is constrained to a narrow Sw-like range (∼45%–46%), whereas the Nf-HNS transition spans a broader interval (∼7.0%–14.4%). This aligns with the empirically defined ≥15% tissue criterion for manual HNS classification (Table 1) and approximates the observed c1 range. In terms of clinical reliability, one would critically assess such a threshold, re-examine the material, and use other methods for further validation to establish certainty. If this fits within a clinical context, however, the indication that an HNS diagnosis is most likely here would be extremely significant. Furthermore, the knowledge of the potential small size of the areas is valuable as a result of the study's certainty.

### Artifact effects and annotation quality

Training-test artifacts mismatch represents a major barrier to generalization. Models may inadvertently exploit technical artifacts. In the training data, the most prominent artifacts were staircase artifacts and pen marks. Sw samples were typically smaller, leading to disproportionately high fractions of patches containing staircase artifacts and background regions. In the HNS cohort, pen marks persisted due to incomplete tissue boundary cropping. In contrast, the test set exhibited a distinct artifact profile, including blur and local scanning defects (Supp. Fig. 6). These findings highlight artifact mismatch as a key driver of performance degradation: models trained on curated datasets may fail when confronted with unseen technical variability. This underscores the need for dedicated preprocessing pipelines to detect and mask artifact regions, as well as for stain- and scanner-robust encoders. Standardized slide preparation and quality control are equally critical, as models may otherwise rely on spurious features rather than tissue morphology. Preparatory analyses further showed that refined pathologist annotations, particularly precise tumor area delineation, substantially improve model performance. Extending annotations (e.g., immune infiltrates) or incorporating additional staining modalities, including immunohistochemistry or specialized stains, may further broaden the applicability of AI-assisted histopathological analysis.

### Limitations

Key limitations include incomplete generalization across staining protocols and scanners, artifact-related performance degradation, and the risk of cohort-specific overfitting. The multicenter design introduced valuable heterogeneity, but also center- and scanner-specific confounders that could not be fully balanced across validation folds because HNS is rare and cases were concentrated in expert referral centers. As a result, some performance may still reflect acquisition- or institution-specific characteristics, despite strict patient-level separation and fold-wise preprocessing, and the cohort reflects real-world expert case availability rather than a fully factorial design. Although two scanning systems were included, residual scanner effects cannot be excluded. Therefore, our findings should be interpreted primarily as evidence of feasibility and performance in an expert-curated multicenter setting. The sample size remains modest for modern DL standards, increasing the likelihood that some models overfit subtle cohort-specific patterns. Whereas we did not observe a typical overfitting profile and applied standard safeguards (patient-level splits, normalization), only evaluation on additional, fully independent external cohorts will conclusively rule out overfitting and confirm robustness across labs and scanners. Methodologically, our approach presupposes the presence of both Sw- and Nf-like components on the slide. HNS cases in which only one component is sampled are at risk of misclassification, and in such scenarios, molecular techniques, including methylation profiling, and clinical context remain essential. From a pathological perspective, the models are deliberately narrow in scope and cannot be expected to generalize to other PNST entities or histological mimics without additional training or fine-tuning. Their outputs should therefore be viewed as a “second reader” for the HNS–Nf–Sw differential rather than as a comprehensive PNST classifier.

### Clinical implications and future directions

Finally, benchmarking against interobserver variability to confirm clinical utility and generalizability is very important, and the development of precise criteria (as we have done in this study with the five main criteria in Table 1) is a key factor in ensuring success; this requires numerous team meetings, consultations with experts and in-depth discussions of errors, sometimes involving difficult specimens that have also had to be re-evaluated over the years. Thus, benchmarking against interobserver variability will be crucial to establish clinical utility. What insights can be gained from the study to help general practicing pathologists, bone and soft tissue pathologists or neuropathologists make the correct diagnosis? The main insight is that AI can provide reliable, task-specific support for recognizing HNS in challenging PNST cases, but should be used in a human-in-the-loop fashion as a decision-support tool rather than an autonomous diagnostic system. Then, AI can provide valuable support here in diagnosing HNS.

## Conclusion

This study shows that HNS can be reliably distinguished from conventional schwannoma and neurofibroma using a compact, interpretable AI workflow that combines foundation encoders, MIL models, and a patch-level classifier operating on routine H&E slides. MIL models based on UNI and ResNet50 achieved near-perfect internal three-class performance and high external HNS accuracy, whereas patch-level classifiers with CONCH or ResNet50 embeddings provided robust probability maps and supported data-driven thresholds for the proportion of Sw- and Nf-like tissue required to classify a case as HNS. Stain-normalized preprocessing generally improved or stabilized performance for foundation encoders, though optimal normalization was model dependent, underscoring the need to tune preprocessing to the encoder and deployment environment rather than applying a one-size-fits-all strategy. In practical terms, the combination of stain-normalized inputs, interpretable heatmaps, and transparent thresholds allows pathologists to quantify and visualize hybrid components, standardize reports, and triage borderline cases for additional review or molecular work-up. Larger, multicenter validation studies and prospective human-in-the-loop evaluations are now needed to confirm generalizability, benchmark against interobserver variability, and establish this approach as a clinically reliable decision-support tool in PNST diagnostics.

## Ethics statement and patient consent

The study was reported to and approved by the local ethics committee of the state chamber of physicians (Landesärztekammer) of Rhineland-Palatinate (no. 2024-17488). Published research complies with the guidelines for human studies and was conducted ethically in accordance with the World Medical Association Declaration of Helsinki.

## CRediT authorship contribution statement

M.A. organized the entire study under the supervision of A.H. to obtain a doctoral degree in medicine, therefore the data generated by him are part of his doctoral thesis (dissertation). F.H. created and implemented computational experiments based on preliminary experiments by P.S., and performed statistical analyses. A.H, F.H., and M.A. wrote the manuscript. F.H., who supervised the bioinformatic approach, will use his contribution as part of his doctoral thesis (dissertation) to obtain a doctoral degree. C.K. neuropathologically evaluated tumors entering the analysis. A.H. (neuropathologist) and M.A. selected the specimens for analysis. D.T. took responsibility for figures, references, and supported manuscript writing. A.H. was responsible for the entire scientific approach and revised the manuscript in the end. All contributors have contributed to the manuscript and revised the final version.

## Funding

The study was not financially supported. This work discloses support by the 10.13039/100000002National Institutes of Health, the Welcome Trust, and the 10.13039/100000011Howard Hughes Medical Institute.

## Declaration of competing interest

The authors declare no competing interests.

## Data Availability

The datasets generated or analyzed are available from the corresponding author upon reasonable request. The Source Code is made publicly available upon publication: https://github.com/hcmlab/AIDD-HNS.

## Appendix A Supplementary data

Supplementary material 1: Supplementary Figures 1-8

Supplementary material 2: Supplemetary Tables 1-3
