# Supplementary material for: Foundation artificial intelligence models enable high-accuracy diagnostic differentiation of hybrid neurofibroma/schwannoma using whole-slide images
Source: J Pathol Inform. 2026 Jul 28;22:100700. doi: 10.1016/j.jpi.2026.100700 (PMC13524541; doi:10.1016/j.jpi.2026.100700)

Supplementary Figures

Foundation Models Enable High-Accuracy Diagnostic Differentiation of Hybrid Neurofibroma/Schwannoma Using Whole-Slide Images

Fabio Hellmann, Maxim Anokhin, Pascal Schimmler, Daniel Tippner, Stefan Plontke, Catena Kresbach, Martin Mensah, Elisabeth André, and Anja Harder

Supplementary Figure 1

ROI selection of neurofibroma-like (a) and schwannoma-like (b) tissue.

a)

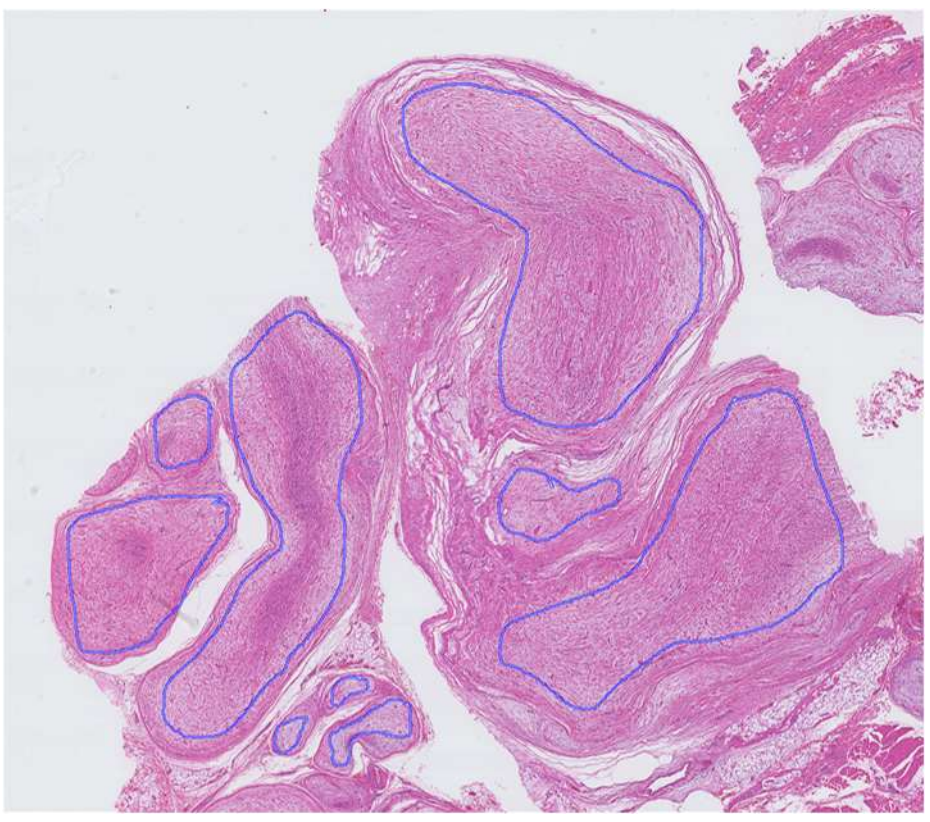

b)

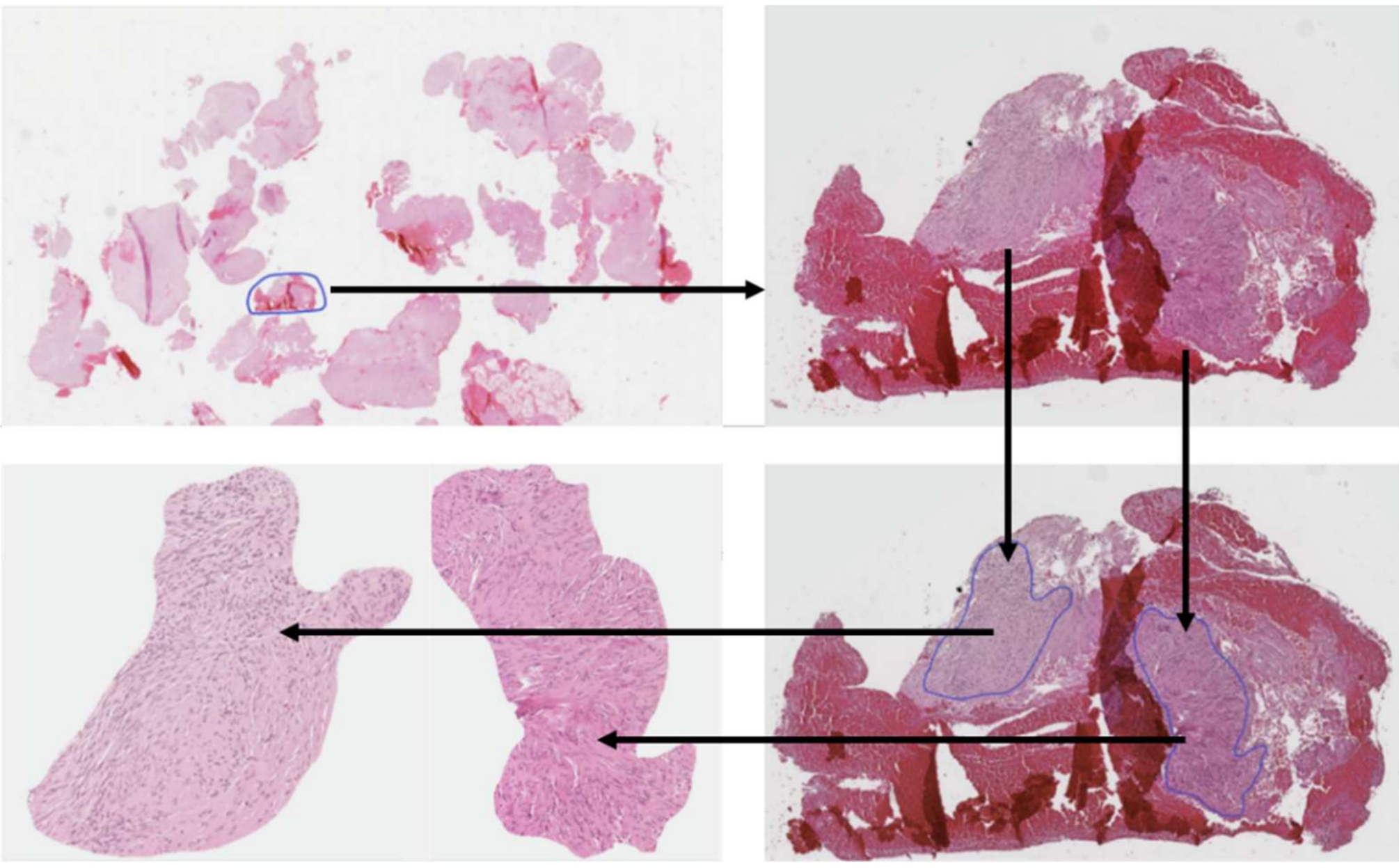

Manual ROI selection is exemplarily shown for several areas of a neurofibroma (a) and as an example for a schwannoma (often resection and embedded in small pieces) (b) to avoid artefacts, bleedings, etc., for the DL process.

Supplementary Figure 2

Example of manually assisted ROI selection of tissue of a hybrid neurofibroma / schwannoma.

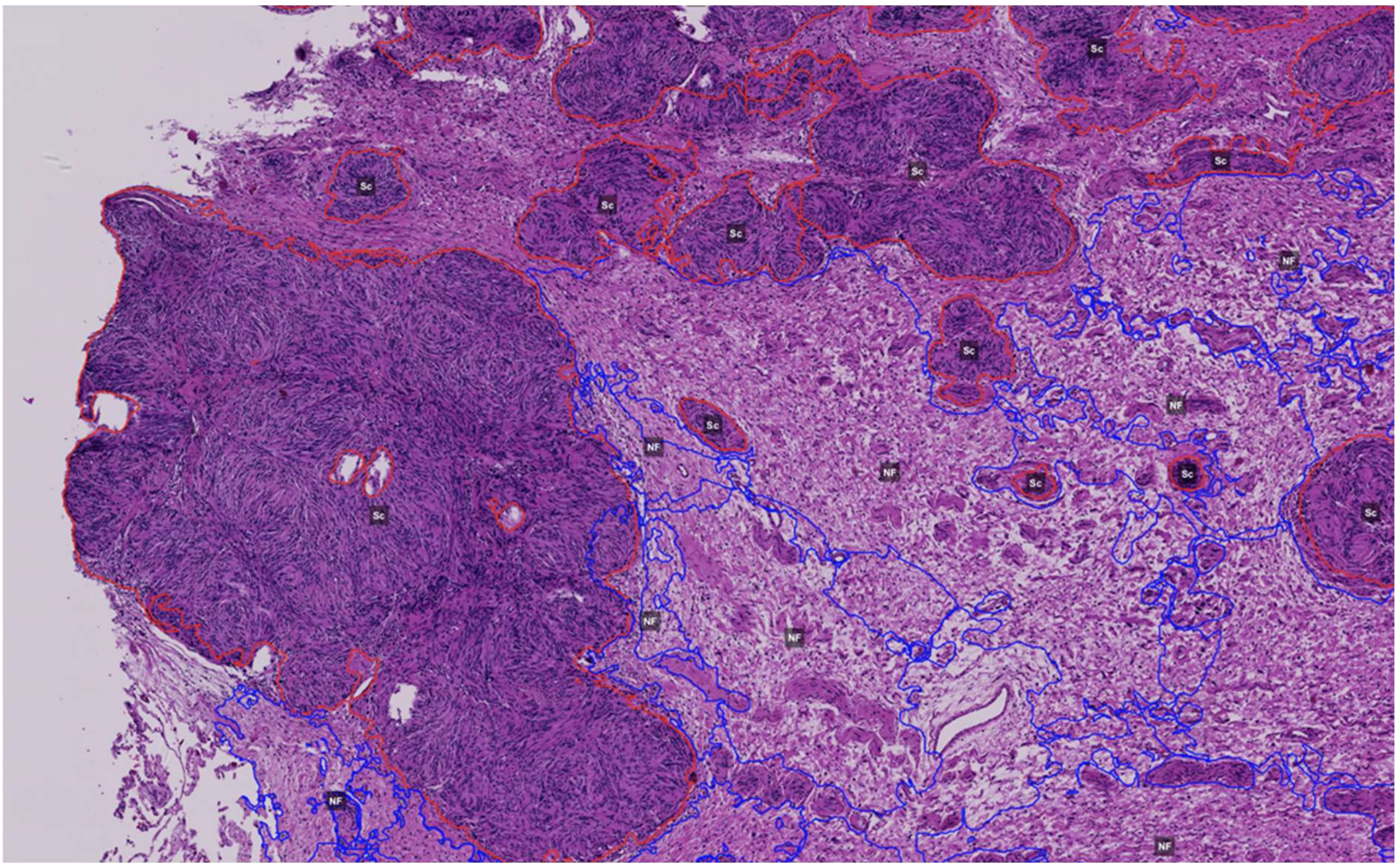

Supplementary Figure 3

Overview of multiple instance learning (MIL) algorithms.

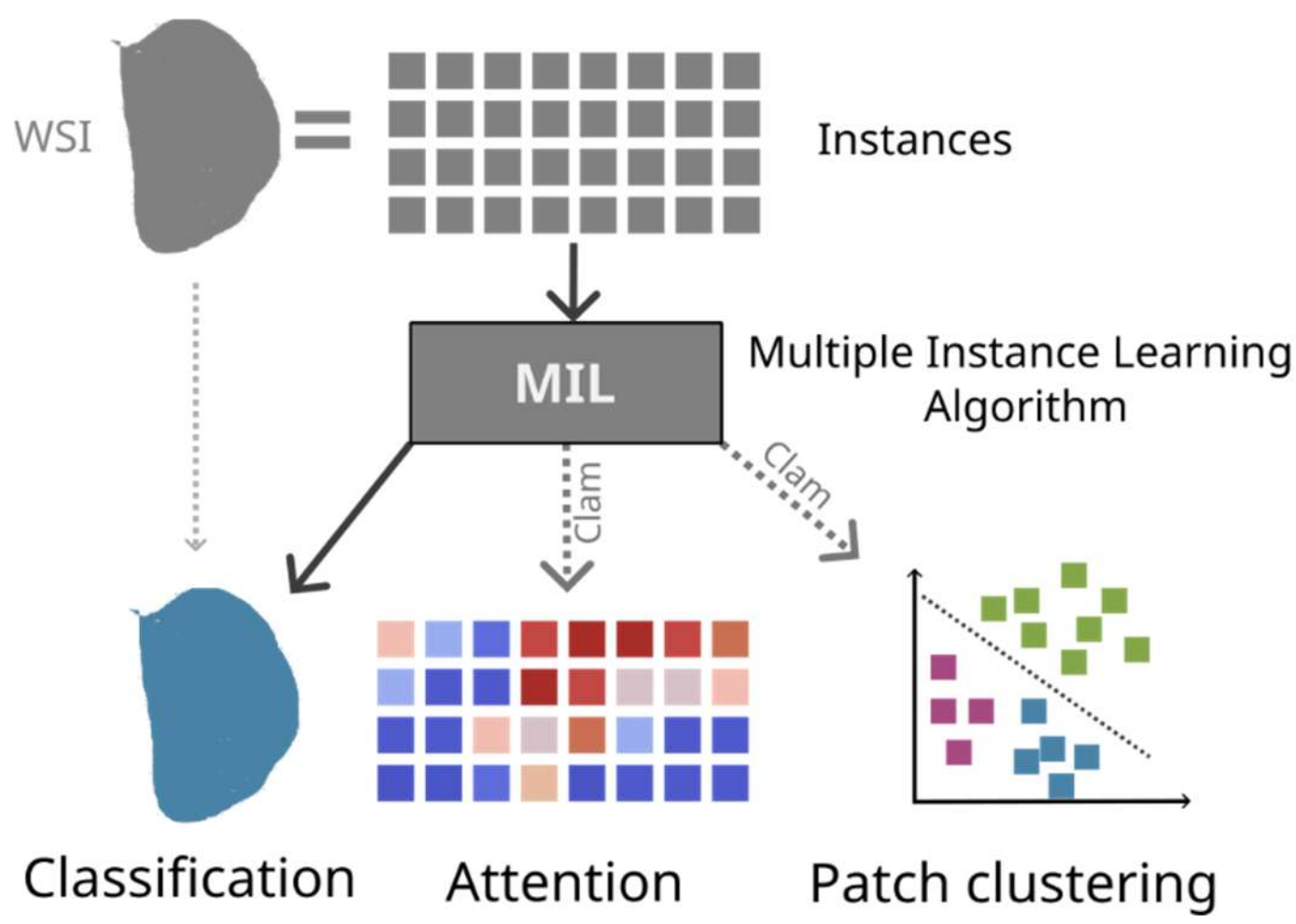

Training of three bag-level MIL algorithms that can take in all embeddings from one WSI at once to predict its class. As a baseline, a simple feedforward network (mMIL) trained under the standard MIL assumption and adapted to the multiclass problem was used.

Supplementary Figure 4

Patch Classifier pipeline.

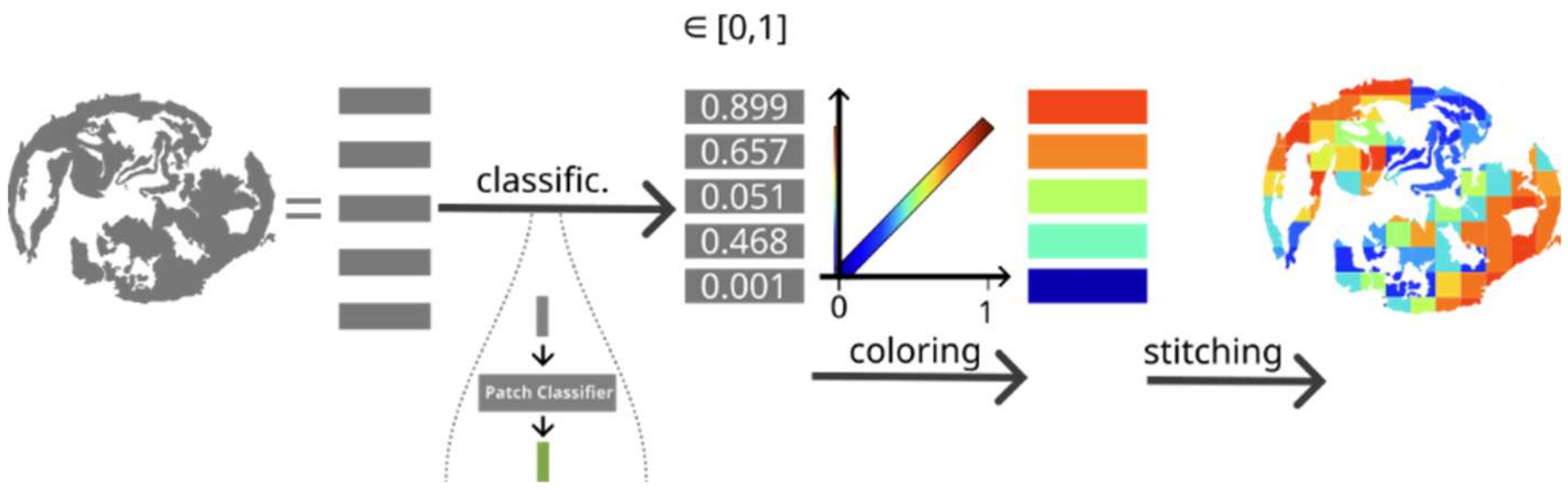

Visualization after stitching identifies schwannoma and neurofibroma regions.

Supplementary Figure 5

Ground truth segmentation of hybrid neurofibroma / schwannoma (HNS), attention of schwannoma, and overlayed Patch classification.

a)

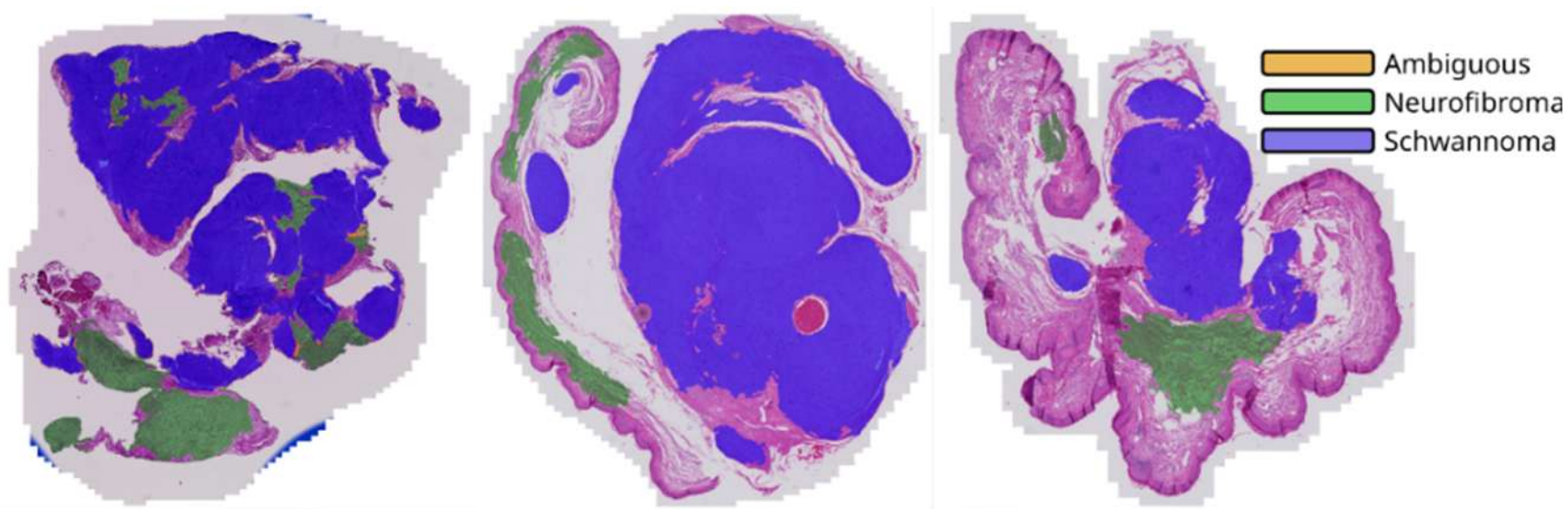

b)

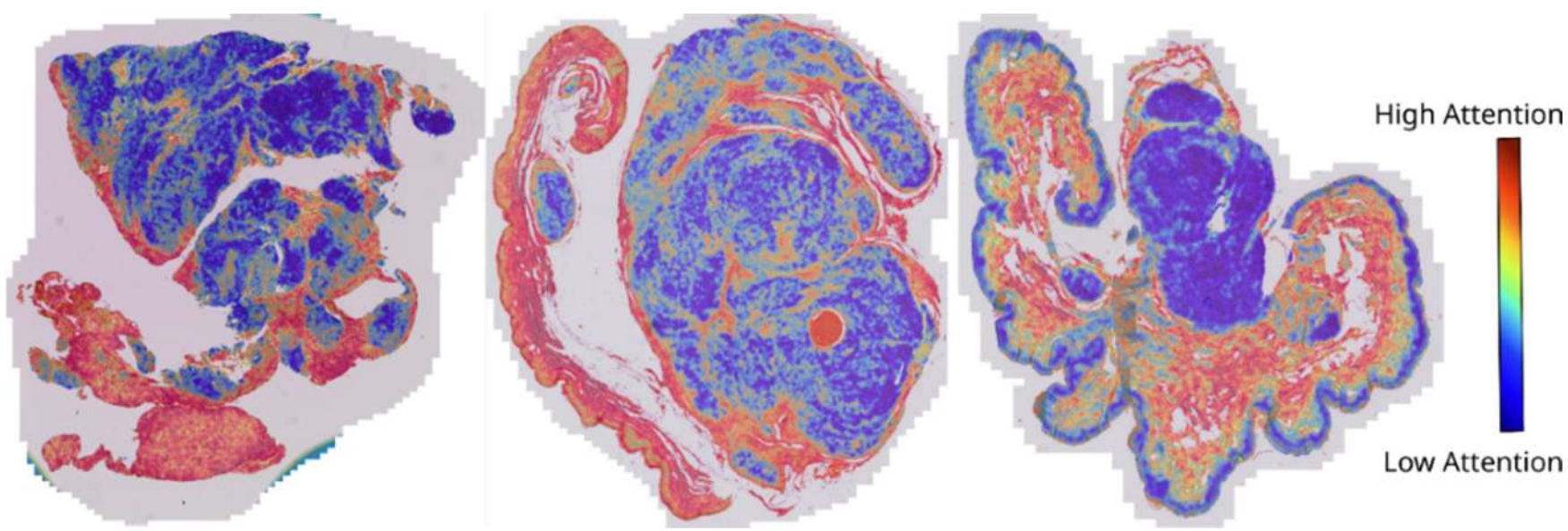

c)

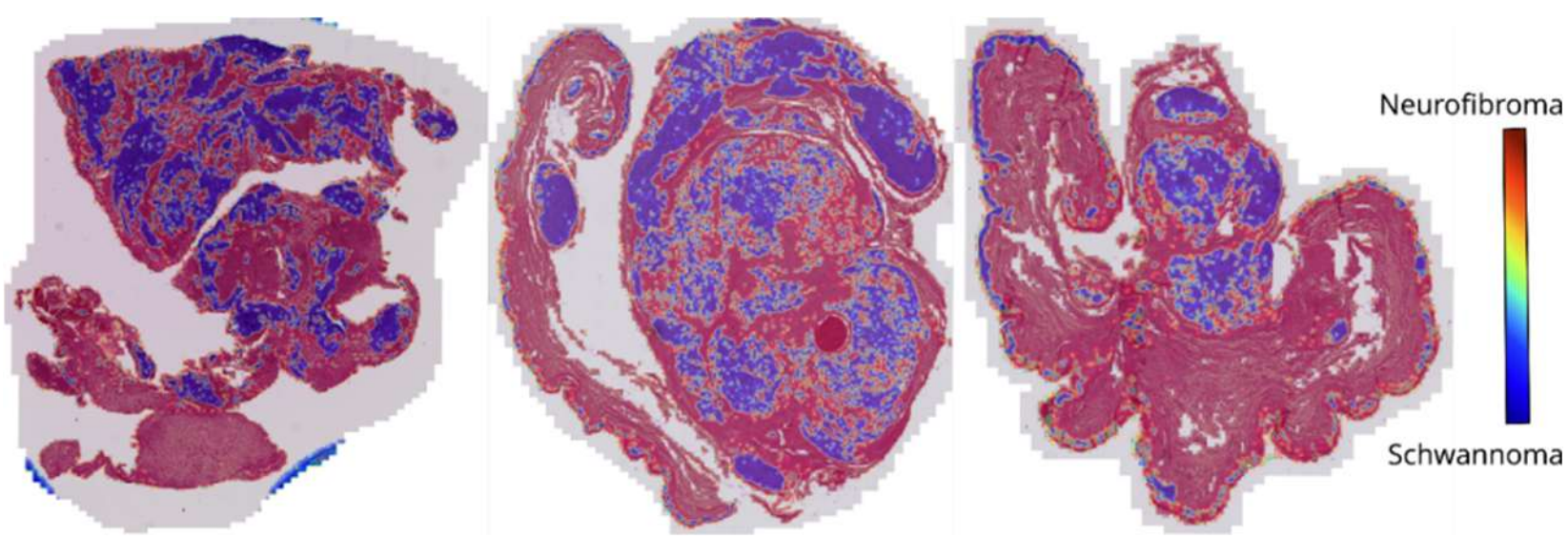

a) Groundtruth segmentation of different hybrid neurofibroma / schwannoma (HNS) tumor parts were established manually. b) Attention of the Schwannoma branch over a sample of the held-out hybrid neurofibroma/ schwannoma (HNS). According to the colour coding, high attention indicates neurofibroma (Nf). c) Patch classification overlayed over a sample of the held-out hybrid neurofibroma/ schwannoma (HNS).

Supplementary Figure 6

Artifacts identified affecting model performance and generalization and exceedingly precise exclusion of areas such as Antoni B areas in Schwannoma potentially distorting the learning process.

a)

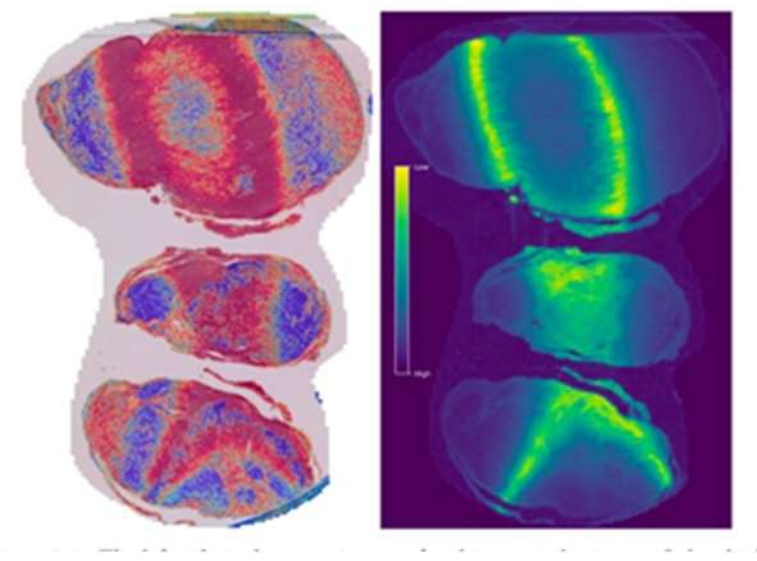

The left side: attention map. It has high attention in a suspicious pattern and under closer inspection, the WSI is blurred in large areas. To better quantify this observation, we estimate the entropy, by patching the image, compressing it to JPEG and plotting the size in bytes. The low entropy areas correspond with the high attention regions.

b)

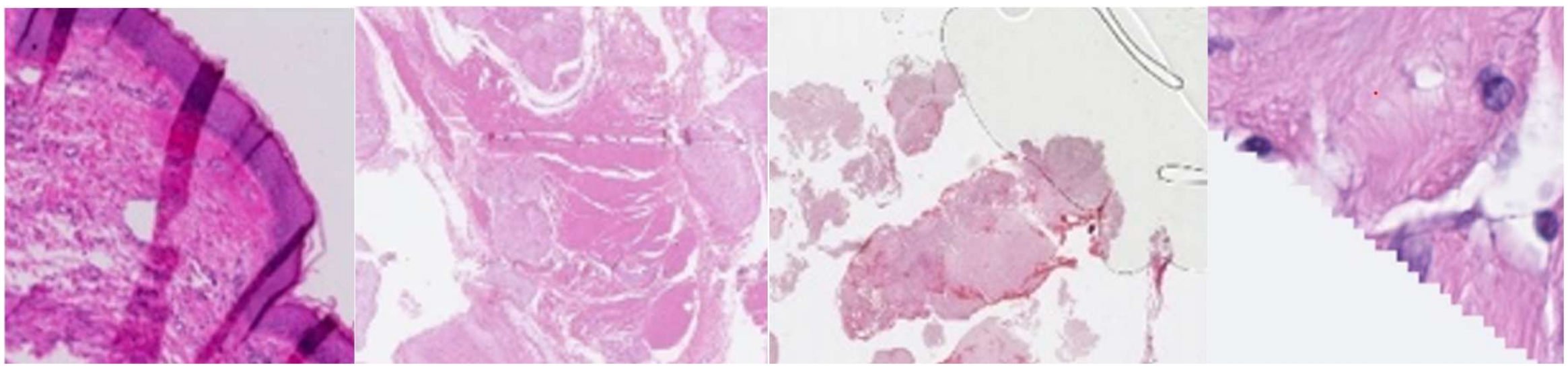

Folded tissue, tissue with scratch marks, air bubbles, and staircase effects (from left to right).

c)

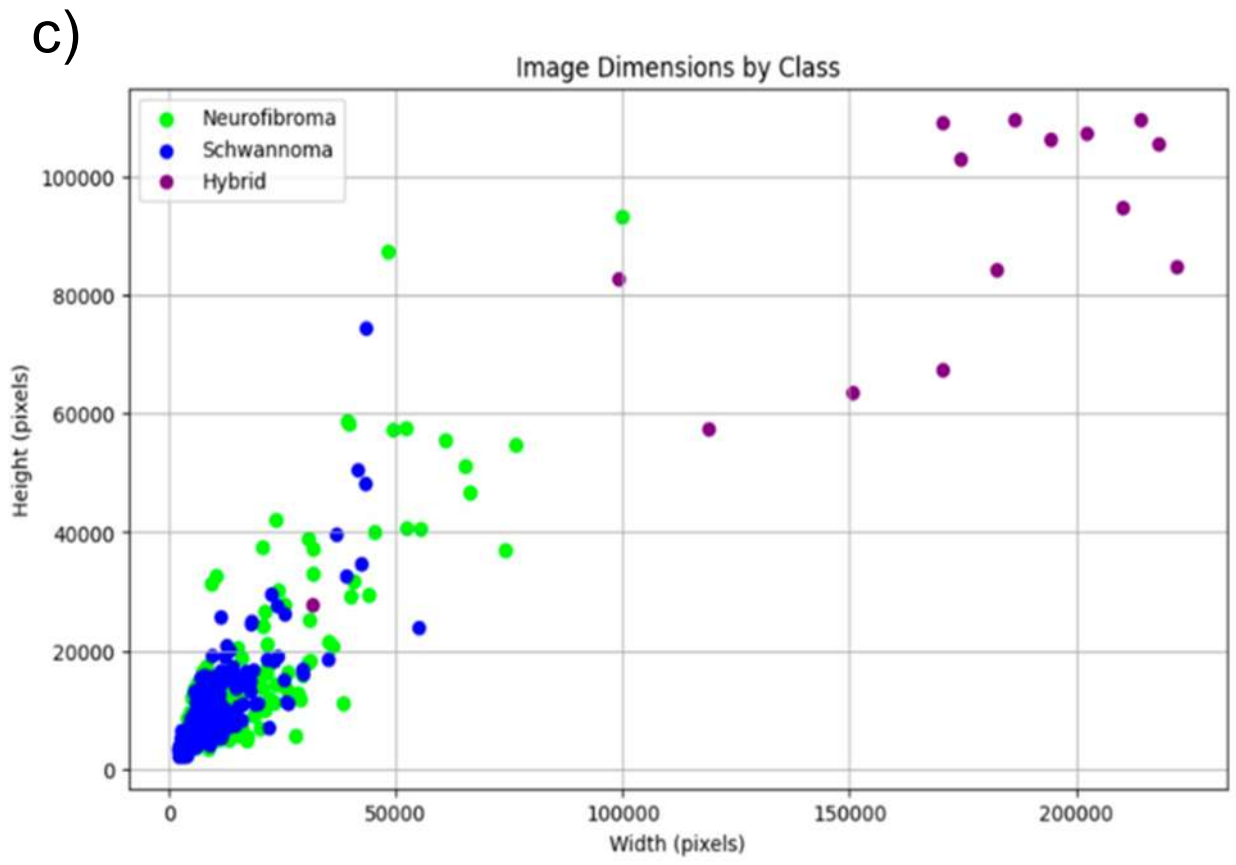

Range of WSI sizes of selected tumors.

d)

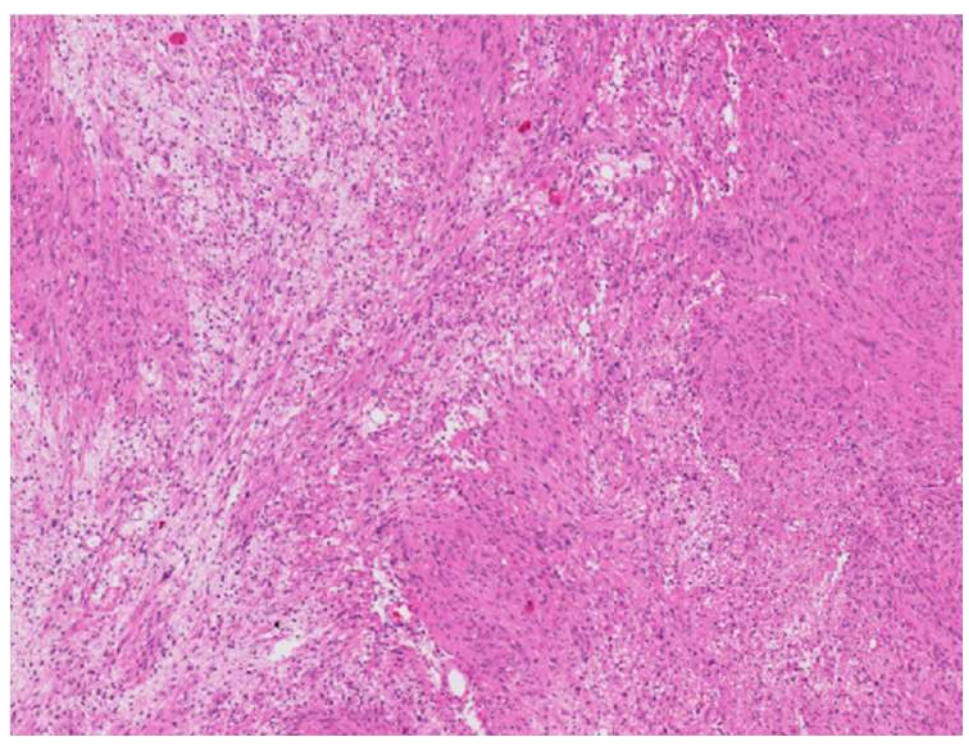

Discriminative exclusion Antoni B areas.

Supplementary Figure 7

The confusion matrices for each fold on the validation set of the best performing MIL-based model – the mCLAM with UNI encoder and stain normalization.

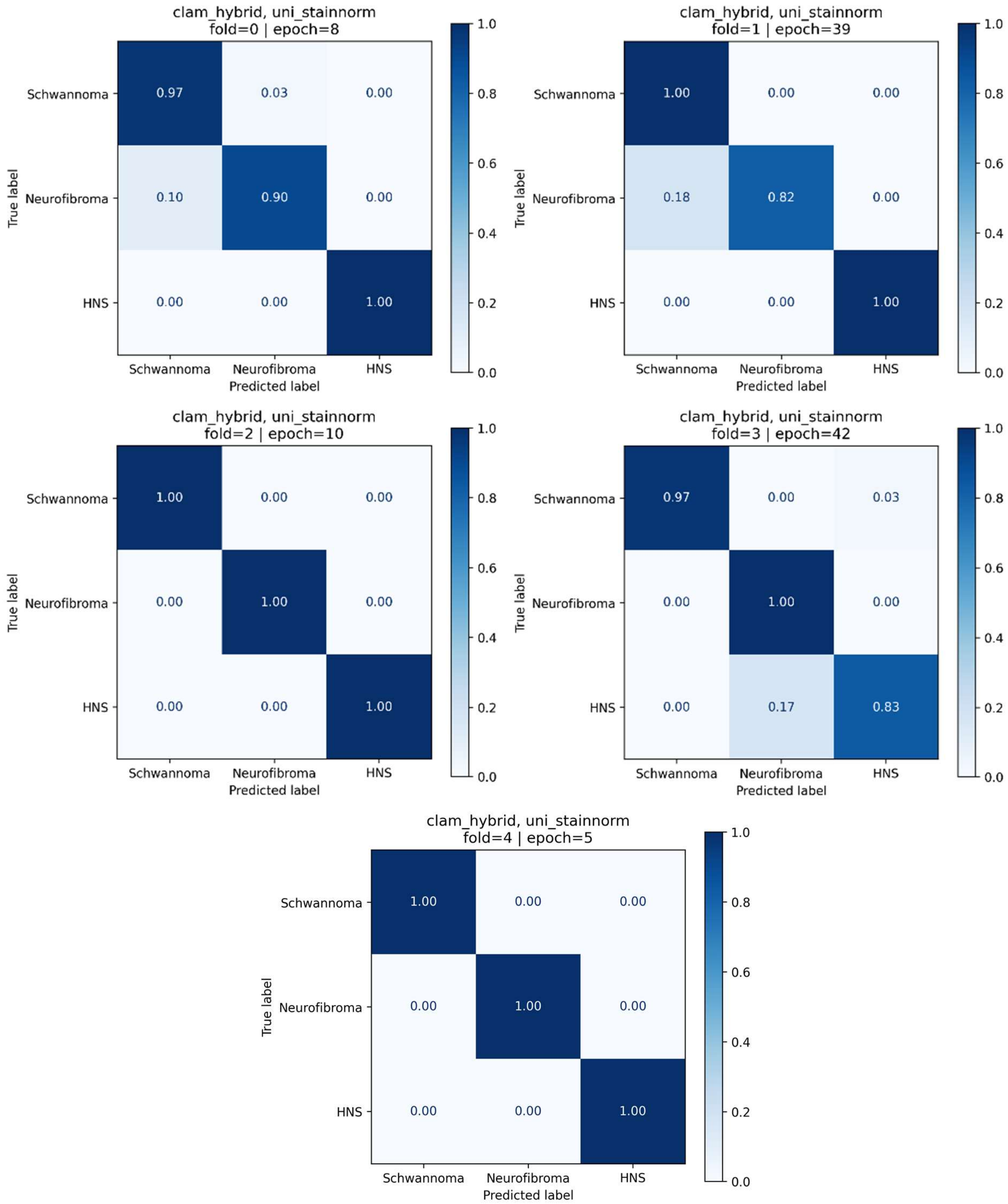

Supplementary Figure 8

The confusion matrices for each fold on the validation set of the best performing Patch-based model using the UNI encoder and stain normalization.

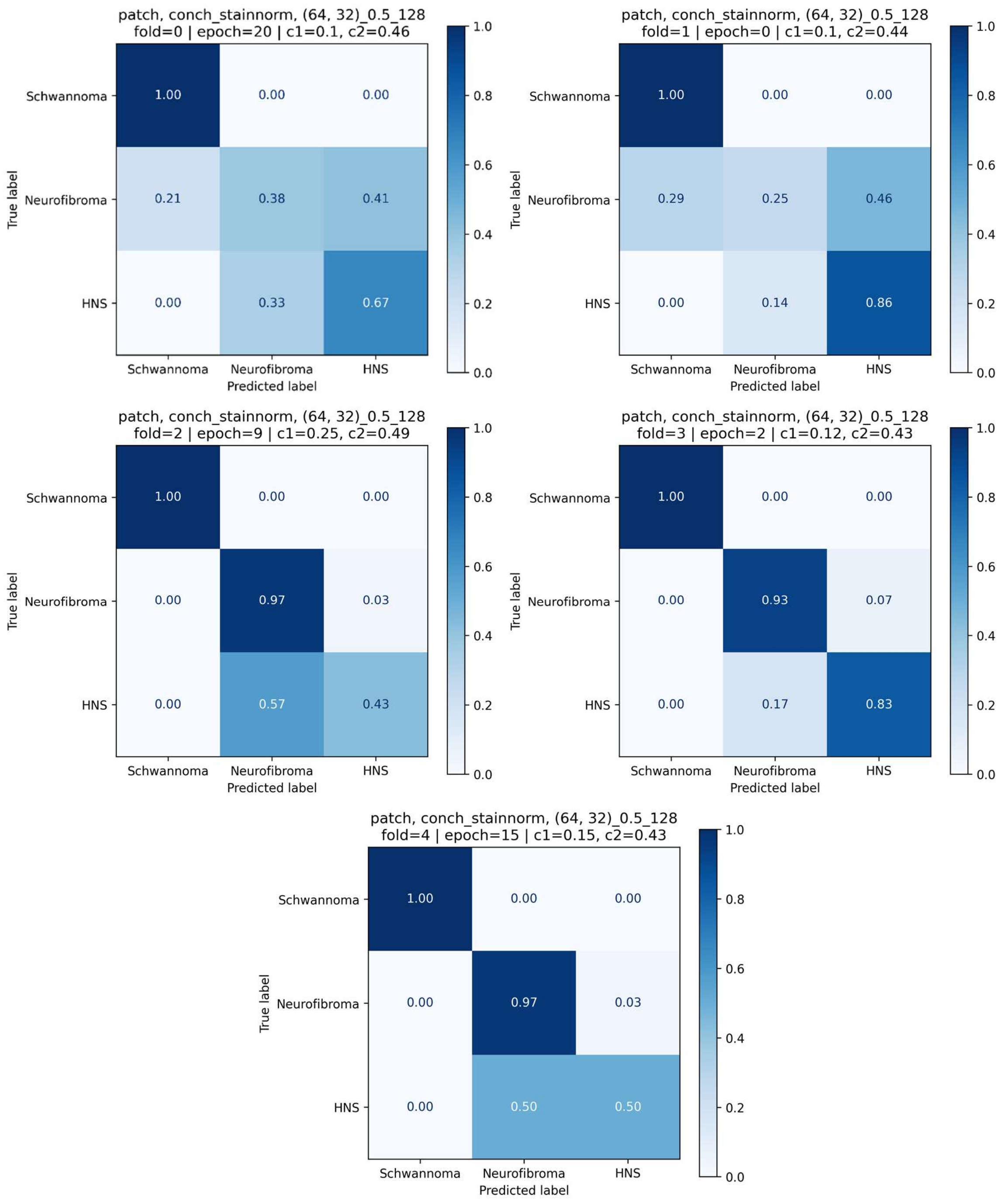

Supplement: Supplementary file 1 [file mmc1.pdf]
