# Supplementary material for: Foundation artificial intelligence models enable high-accuracy diagnostic differentiation of hybrid neurofibroma/schwannoma using whole-slide images
Source: J Pathol Inform. 2026 Jul 28;22:100700. doi: 10.1016/j.jpi.2026.100700 (PMC13524541; doi:10.1016/j.jpi.2026.100700)
Supplement: Supplementary file 2 [file mmc2.pdf]

Supplementary Tables

Foundation Models Enable High-Accuracy Diagnostic Differentiation of Hybrid Neurofibroma/Schwannoma Using Whole-Slide Images

Fabio Hellmann, Maxim Anokhin, Pascal Schimmler, Daniel Tippner, Stefan Plontke, Catena Kresbach, Martin Mensah, Elisabeth André, and Anja Harder

Supplementary Table 1

Validation results for the mMIL, CLAM, and mCLAM classifiers with UNI, ResNet50, and Conch encoder.

The metrics display the 5-Fold CV mean, with their respective standard deviations and 95% confidence intervals, based on the validation set.

Model	Encoder	Norm.	MCC	micro F1	macro AUROC	macro Accuracy	Precision Sw	Precision Nf	Precision HNS	Recall Sw	Recall Nf	Recall HNS
mCLAM	UNI	stainnorm	**0.95±0.05 [0.90,0.99]**	**0.97±0.03 [0.94,1.00]**	**1.00±0.00 [1.00,1.00]**	0.97±0.03 [0.94,0.99]	0.96±0.05	**0.99±0.02**	0.97±0.07	**0.99±0.01**	0.94±0.08	0.97±0.07
CLAM	UNI	stainnorm	0.94±0.08 [0.87,1.01]	0.96±0.05 [0.92,1.00]	1.00±0.00 [0.99,1.00]	0.94±0.05 [0.90,0.99]	0.98±0.05	0.98±0.02	0.86±0.14	**0.99±0.01**	0.93±0.12	0.91±0.08
CLAM	UNI	gray	0.94±0.08 [0.87,1.01]	0.96±0.05 [0.92,1.01]	1.00±0.00 [0.99,1.00]	0.96±0.04 [0.92,1.00]	0.96±0.07	0.98±0.03	0.93±0.10	0.99±0.02	0.92±0.14	0.97±0.07
mCLAM	UNI	default	0.94±0.05 [0.90,0.98]	**0.97±0.03 [0.94,0.99]**	**1.00±0.00 [1.00,1.00]**	**0.97±0.02 [0.95,0.98]**	**0.98±0.02**	0.97±0.04	0.90±0.14	0.98±0.03	0.94±0.05	**0.97±0.06**
CLAM	UNI	default	0.90±0.11 [0.80,0.99]	0.94±0.07 [0.88,0.99]	**1.00±0.00 [1.00,1.00]**	0.94±0.06 [0.89,0.99]	0.95±0.09	0.97±0.04	0.86±0.19	0.98±0.03	0.87±0.21	**0.97±0.06**
mCLAM	UNI	gray	0.90±0.08 [0.83,0.97]	0.94±0.06 [0.89,0.99]	**1.00±0.00 [1.00,1.00]**	0.94±0.04 [0.90,0.98]	0.97±0.03	**0.99±0.02**	0.77±0.23	**0.99±0.01**	0.85±0.15	**0.97±0.06**
mCLAM	CONCH	gray	0.89±0.08 [0.82,0.95]	0.93±0.05 [0.89,0.97]	0.99±0.02 [0.97,1.00]	0.93±0.02 [0.92,0.95]	0.98±0.03	0.92±0.08	0.86±0.18	0.94±0.07	0.92±0.08	0.94±0.09
CLAM	CONCH	gray	0.89±0.06 [0.84,0.94]	0.93±0.04 [0.90,0.97]	0.99±0.01 [0.98,1.00]	0.92±0.02 [0.90,0.94]	0.98±0.05	0.89±0.07	**0.97±0.06**	0.93±0.09	**0.96±0.08**	0.88±0.14

CLAM	CONCH	stainnorm	0.87±0.09 [0.80,0.95]	0.93±0.05 [0.88,0.97]	0.99±0.01 [0.97,1.00]	0.90±0.07 [0.85,0.96]	0.97±0.06	0.90±0.07	0.89±0.18	0.95±0.06	0.91±0.10	0.85±0.16
CLAM	CONCH	default	0.86±0.11 [0.76,0.96]	0.92±0.07 [0.85,0.98]	0.99±0.01 [0.98,1.00]	0.91±0.08 [0.85,0.98]	0.94±0.10	0.93±0.07	0.82±0.19	0.96±0.05	0.85±0.19	0.93±0.17
CLAM	ResNet50	default	0.86±0.11 [0.76,0.95]	0.92±0.06 [0.86,0.97]	0.98±0.03 [0.95,1.00]	0.90±0.06 [0.85,0.95]	0.95±0.06	0.91±0.09	0.88±0.16	0.94±0.08	0.89±0.13	0.88±0.14
mCLAM	CONCH	default	0.86±0.10 [0.77,0.95]	0.91±0.07 [0.85,0.97]	0.98±0.02 [0.97,1.00]	0.92±0.06 [0.87,0.98]	0.96±0.10	0.93±0.07	0.80±0.23	0.95±0.06	0.84±0.19	**0.97±0.06**
mMIL	UNI	default	0.86±0.08 [0.79,0.93]	0.92±0.04 [0.88,0.96]	0.96±0.05 [0.91,1.01]	0.92±0.05 [0.88,0.96]	0.85±0.18	0.95±0.03	0.89±0.12	0.95±0.04	0.87±0.13	0.93±0.09
mCLAM	CONCH	stainnorm	0.86±0.08 [0.79,0.93]	0.92±0.05 [0.87,0.96]	0.98±0.03 [0.95,1.00]	0.89±0.06 [0.84,0.94]	0.97±0.04	0.90±0.08	0.81±0.21	0.93±0.09	0.92±0.11	0.83±0.16
mCLAM	ResNet50	stainnorm	0.84±0.08 [0.77,0.91]	0.91±0.05 [0.87,0.95]	0.98±0.02 [0.96,0.99]	0.89±0.02 [0.87,0.90]	0.93±0.07	0.89±0.04	0.88±0.12	0.93±0.04	0.88±0.12	0.85±0.16
mCLAM	ResNet50	default	0.84±0.06 [0.78,0.89]	0.90±0.04 [0.87,0.94]	0.98±0.01 [0.97,0.99]	0.86±0.05 [0.81,0.90]	0.94±0.04	0.92±0.07	0.83±0.23	0.95±0.08	0.87±0.15	0.75±0.29
CLAM	ResNet50	gray	0.82±0.12 [0.71,0.92]	0.89±0.07 [0.83,0.95]	0.97±0.02 [0.96,0.99]	0.88±0.05 [0.83,0.93]	0.94±0.10	0.89±0.06	0.81±0.18	0.94±0.04	0.82±0.22	0.87±0.14
CLAM	ResNet50	stainnorm	0.81±0.13 [0.70,0.93]	0.89±0.07 [0.83,0.95]	0.97±0.03 [0.94,1.00]	0.85±0.08 [0.78,0.92]	0.93±0.08	0.87±0.09	0.86±0.19	0.94±0.05	0.85±0.14	0.76±0.16
mCLAM	ResNet50	gray	0.77±0.11 [0.67,0.87]	0.86±0.07 [0.80,0.92]	0.97±0.02 [0.95,0.98]	0.83±0.07 [0.77,0.90]	0.92±0.12	0.87±0.07	0.73±0.18	0.93±0.07	0.78±0.23	0.79±0.20
mMIL	CONCH	default	0.75±0.07 [0.68,0.81]	0.88±0.04 [0.84,0.92]	0.96±0.02 [0.94,0.97]	0.81±0.06 [0.76,0.87]	0.84±0.11	0.89±0.05	0.76±0.19	0.92±0.04	0.82±0.24	0.71±0.23
mMIL	UNI	stainnorm	0.74±0.22 [0.55,0.93]	0.84±0.16 [0.70,0.98]	0.91±0.10 [0.83,1.00]	0.87±0.06 [0.81,0.92]	0.73±0.31	0.93±0.04	0.80±0.25	0.93±0.03	0.79±0.22	0.88±0.12
mMIL	UNI	gray	0.73±0.10 [0.64,0.81]	0.86±0.06 [0.80,0.91]	0.93±0.05 [0.89,0.97]	0.85±0.07 [0.79,0.91]	0.83±0.18	0.86±0.12	0.76±0.20	0.84±0.15	0.86±0.16	0.84±0.17
mMIL	CONCH	stainnorm	0.70±0.16 [0.56,0.84]	0.83±0.10 [0.74,0.91]	0.94±0.03 [0.91,0.97]	0.83±0.05 [0.78,0.87]	0.84±0.10	0.90±0.06	0.67±0.31	0.91±0.03	0.79±0.16	0.78±0.15
mMIL	ResNet50	default	0.66±0.13 [0.55,0.77]	0.82±0.04 [0.79,0.85]	0.92±0.07 [0.86,0.97]	0.82±0.08 [0.75,0.88]	0.88±0.09	0.84±0.16	0.59±0.27	0.86±0.05	0.78±0.10	0.81±0.24
mMIL	CONCH	gray	0.62±0.19 [0.45,0.78]	0.77±0.14 [0.65,0.89]	0.91±0.05 [0.86,0.95]	0.78±0.08 [0.71,0.85]	0.71±0.31	0.86±0.09	0.62±0.19	0.88±0.05	0.68±0.24	0.79±0.18
mMIL	ResNet50	gray	0.61±0.07 [0.55,0.67]	0.81±0.03 [0.78,0.83]	0.90±0.05 [0.85,0.95]	0.75±0.06 [0.70,0.81]	0.83±0.05	0.79±0.12	0.62±0.25	0.83±0.08	0.76±0.23	0.66±0.26
mMIL	ResNet50	stainnorm	0.53±0.13 [0.42,0.64]	0.74±0.09 [0.65,0.82]	0.88±0.03 [0.85,0.91]	0.73±0.04 [0.70,0.76]	0.77±0.14	0.76±0.18	0.55±0.30	0.74±0.18	0.78±0.12	0.68±0.24

Supplementary Table 2

Validation results for the Patch classifiers with UNI, ResNet50, and Conch encoder.

The metrics display the 5-Fold CV mean, with their respective standard deviations and 95% confidence intervals, based on the validation set.

Model	Encoder	Normalization	c1	c2	B	D	HS	MCC	micro F1	macro AUROC	macro Accuracy	Precision Sw	Precision Nf	Precision HNS	Recall Sw	Recall Nf	Recall HNS
Patch	CONCH	stainnorm	0.14±0.06 [0.09,0.20]	0.45±0.03 [0.43,0.47]	128	0.5	(64, 32)	0.77±0.18 [0.61,0.93]	0.85±0.12 [0.74,0.96]	0.86±0.08 [0.79,0.92]	0.79±0.10 [0.70,0.87]	0.94±0.08	0.89±0.04	**0.57±0.26**	**1.00±0.00**	0.70±0.35	0.66±0.19
Patch	UNI	stainnorm	0.05±0.02 [0.03,0.06]	0.41±0.06 [0.36,0.46]	64	0.5	(128, 64)	**0.77±0.13 [0.65,0.88]**	**0.85±0.09 [0.78,0.93]**	0.86±0.08 [0.80,0.93]	0.79±0.12 [0.69,0.90]	0.96±0.05	0.92±0.09	0.44±0.21	**1.00±0.00**	0.69±0.22	0.69±0.26
Patch	UNI	stainnorm	0.05±0.01 [0.05,0.06]	0.39±0.03 [0.37,0.42]	128	0.5	(32, 16)	0.76±0.19 [0.59,0.93]	0.85±0.13 [0.73,0.96]	0.86±0.11 [0.76,0.95]	0.78±0.16 [0.64,0.92]	0.96±0.06	0.87±0.15	0.48±0.24	**1.00±0.00**	0.69±0.31	0.66±0.23
Patch	UNI	stainnorm	0.04±0.02 [0.03,0.06]	0.37±0.04 [0.34,0.40]	64	0.5	(32, 16)	0.76±0.17 [0.61,0.90]	0.84±0.13 [0.73,0.95]	0.86±0.10 [0.77,0.94]	0.78±0.14 [0.66,0.91]	0.97±0.04	0.86±0.17	0.43±0.19	**1.00±0.00**	0.66±0.31	0.69±0.19
Patch	UNI	stainnorm	0.04±0.01 [0.03,0.04]	0.42±0.03 [0.39,0.45]	128	0.3	(64, 32)	0.76±0.16 [0.62,0.90]	0.84±0.13 [0.72,0.95]	0.87±0.09 [0.79,0.95]	0.80±0.13 [0.69,0.92]	0.98±0.04	0.88±0.17	0.43±0.19	**1.00±0.00**	0.63±0.32	0.78±0.22
Patch	CONCH	stainnorm	0.13±0.08 [0.05,0.20]	0.48±0.06 [0.42,0.53]	64	0.3	(128, 64)	0.76±0.16 [0.62,0.90]	0.85±0.11 [0.75,0.94]	0.85±0.08 [0.78,0.93]	0.78±0.12 [0.67,0.88]	0.96±0.06	0.89±0.06	0.46±0.26	0.99±0.01	**0.70±0.29**	0.64±0.21
Patch	CONCH	stainnorm	0.07±0.02 [0.05,0.08]	0.45±0.05 [0.41,0.49]	128	0.5	(128, 64)	0.76±0.14 [0.63,0.88]	0.84±0.10 [0.75,0.93]	**0.87±0.07 [0.81,0.93]**	**0.81±0.09 [0.73,0.89]**	0.97±0.06	**0.93±0.06**	0.43±0.19	0.99±0.01	0.64±0.29	**0.79±0.13**
Patch	UNI	gray	0.09±0.06 [0.04,0.15]	0.40±0.07 [0.33,0.46]	64	0.5	(32, 16)	0.75±0.21 [0.56,0.93]	0.83±0.15 [0.70,0.97]	0.86±0.11 [0.76,0.95]	0.79±0.16 [0.65,0.92]	0.96±0.06	0.86±0.16	0.51±0.33	**1.00±0.00**	0.63±0.38	0.73±0.14
Patch	UNI	stainnorm	0.05±0.02 [0.03,0.07]	0.40±0.05 [0.36,0.45]	64	0.5	(64, 32)	0.75±0.18 [0.60,0.91]	0.83±0.14 [0.71,0.96]	0.86±0.09 [0.77,0.94]	0.78±0.13 [0.66,0.90]	0.97±0.04	0.84±0.19	0.45±0.23	**1.00±0.00**	0.64±0.36	0.71±0.10
Patch	UNI	stainnorm	0.04±0.02 [0.02,0.05]	0.38±0.04 [0.34,0.42]	128	0.5	(128, 64)	0.75±0.16 [0.61,0.89]	0.83±0.12 [0.72,0.94]	0.85±0.09 [0.78,0.93]	0.78±0.13 [0.67,0.89]	**0.98±0.03**	0.87±0.14	0.43±0.24	**1.00±0.00**	0.63±0.31	0.70±0.16
Patch	UNI	stainnorm	0.05±0.04 [0.01,0.08]	0.40±0.04 [0.37,0.44]	64	0.3	(64, 32)	0.75±0.15 [0.62,0.88]	0.84±0.10 [0.75,0.93]	0.86±0.09 [0.78,0.93]	0.79±0.12 [0.68,0.90]	0.96±0.05	0.91±0.10	0.41±0.19	**1.00±0.00**	0.64±0.27	0.73±0.25
Patch	UNI	stainnorm	0.05±0.01 [0.03,0.06]	0.40±0.04 [0.37,0.44]	128	0.5	(64, 32)	0.75±0.14 [0.63,0.87]	0.83±0.11 [0.74,0.93]	0.86±0.07 [0.80,0.92]	0.79±0.10 [0.70,0.88]	0.98±0.05	0.90±0.08	0.42±0.22	**1.00±0.00**	0.63±0.28	0.74±0.06
Patch	UNI	stainnorm	0.03±0.02 [0.01,0.04]	0.39±0.06 [0.33,0.45]	128	0.3	(128, 64)	0.74±0.20 [0.56,0.91]	0.81±0.16 [0.67,0.95]	0.85±0.11 [0.75,0.94]	0.77±0.16 [0.63,0.91]	**0.98±0.03**	0.76±0.43	0.43±0.24	**1.00±0.00**	0.58±0.41	0.73±0.20
Patch	UNI	stainnorm	0.03±0.01 [0.02,0.04]	0.39±0.04 [0.36,0.42]	64	0.3	(32, 16)	0.74±0.17 [0.59,0.89]	0.83±0.13 [0.71,0.94]	0.86±0.10 [0.77,0.94]	0.79±0.15 [0.66,0.92]	0.97±0.04	0.86±0.21	0.42±0.23	**1.00±0.00**	0.61±0.32	0.76±0.16

Patch	UNI	stainnorm	0.04±0.02 [0.02,0.06]	0.41±0.06 [0.35,0.46]	64	0.3	(128, 64)	0.74±0.17 [0.59,0.88]	0.82±0.13 [0.70,0.94]	0.86±0.10 [0.77,0.94]	0.78±0.15 [0.65,0.92]	**0.98±0.03**	0.85±0.25	0.39±0.19	**1.00±0.00**	0.60±0.33	0.76±0.21
Patch	CONCH	stainnorm	0.07±0.02 [0.05,0.09]	0.43±0.06 [0.38,0.48]	128	0.3	(128, 64)	0.74±0.15 [0.61,0.88]	0.83±0.11 [0.74,0.93]	0.86±0.07 [0.79,0.92]	0.79±0.10 [0.70,0.88]	0.95±0.06	0.92±0.07	0.44±0.24	0.99±0.01	0.64±0.30	0.74±0.18
Patch	UNI	gray	0.06±0.05 [0.02,0.11]	0.44±0.11 [0.34,0.53]	128	0.3	(64, 32)	0.73±0.23 [0.53,0.94]	0.82±0.16 [0.68,0.97]	0.84±0.12 [0.74,0.95]	0.77±0.18 [0.61,0.92]	0.95±0.07	0.84±0.23	0.51±0.34	**1.00±0.00**	0.62±0.41	0.68±0.26
Patch	UNI	gray	0.07±0.04 [0.04,0.11]	0.40±0.11 [0.30,0.50]	128	0.5	(64, 32)	0.73±0.23 [0.53,0.93]	0.83±0.15 [0.70,0.96]	0.84±0.12 [0.73,0.95]	0.76±0.18 [0.61,0.92]	0.93±0.07	0.83±0.23	0.49±0.33	**1.00±0.00**	0.64±0.37	0.64±0.24
Patch	UNI	gray	0.02±0.01 [0.02,0.03]	0.40±0.08 [0.33,0.47]	128	0.5	(128, 64)	0.73±0.18 [0.57,0.88]	0.80±0.14 [0.68,0.93]	0.85±0.09 [0.77,0.93]	0.78±0.14 [0.66,0.90]	0.97±0.04	0.90±0.17	0.43±0.34	**1.00±0.00**	0.55±0.36	0.79±0.24
Patch	UNI	default	0.04±0.03 [0.02,0.06]	0.33±0.09 [0.25,0.41]	128	0.5	(128, 64)	0.73±0.17 [0.58,0.88]	0.83±0.11 [0.74,0.93]	0.85±0.09 [0.76,0.93]	0.78±0.14 [0.66,0.89]	0.92±0.09	0.89±0.11	0.46±0.22	**1.00±0.00**	0.65±0.27	0.68±0.26
Patch	CONCH	stainnorm	0.08±0.02 [0.06,0.10]	0.45±0.02 [0.44,0.47]	64	0.5	(64, 32)	0.73±0.12 [0.63,0.83]	0.83±0.08 [0.75,0.90]	0.85±0.07 [0.79,0.91]	0.77±0.10 [0.69,0.85]	0.97±0.05	0.89±0.05	0.35±0.13	**1.00±0.00**	0.64±0.23	0.67±0.21
Patch	ResNet50	stainnorm	0.14±0.11 [0.04,0.24]	0.46±0.06 [0.41,0.51]	64	0.5	(32, 16)	0.72±0.21 [0.53,0.90]	0.83±0.14 [0.70,0.95]	0.82±0.12 [0.71,0.93]	0.72±0.18 [0.57,0.88]	0.94±0.09	0.81±0.14	0.38±0.34	0.99±0.01	0.69±0.32	0.48±0.32
Patch	ResNet50	stainnorm	0.11±0.02 [0.09,0.13]	0.49±0.08 [0.42,0.56]	64	0.5	(64, 32)	0.72±0.21 [0.53,0.90]	0.81±0.15 [0.68,0.95]	0.84±0.10 [0.75,0.93]	0.76±0.14 [0.64,0.89]	0.94±0.09	0.84±0.20	0.44±0.26	0.99±0.01	0.61±0.41	0.69±0.28
Patch	UNI	gray	0.04±0.03 [0.02,0.07]	0.41±0.07 [0.36,0.47]	128	0.3	(128, 64)	0.72±0.21 [0.53,0.90]	0.81±0.15 [0.69,0.94]	0.84±0.11 [0.74,0.94]	0.76±0.16 [0.61,0.90]	0.95±0.07	0.84±0.23	0.41±0.20	**1.00±0.00**	0.60±0.37	0.68±0.26
Patch	UNI	stainnorm	0.06±0.04 [0.02,0.10]	0.42±0.07 [0.36,0.48]	128	0.3	(32, 16)	0.72±0.20 [0.54,0.90]	0.82±0.14 [0.71,0.94]	0.84±0.12 [0.74,0.94]	0.76±0.17 [0.62,0.91]	0.95±0.07	0.83±0.23	0.40±0.20	**1.00±0.00**	0.62±0.32	0.67±0.22
Patch	UNI	gray	0.05±0.05 [0.01,0.09]	0.47±0.14 [0.35,0.59]	64	0.3	(64, 32)	0.72±0.20 [0.54,0.89]	0.80±0.15 [0.67,0.94]	0.83±0.11 [0.74,0.93]	0.75±0.17 [0.60,0.90]	0.97±0.06	0.83±0.23	0.42±0.28	**1.00±0.00**	0.57±0.39	0.68±0.26
Patch	ResNet50	stainnorm	0.15±0.11 [0.05,0.25]	0.48±0.06 [0.43,0.53]	128	0.3	(32, 16)	0.72±0.19 [0.56,0.88]	0.82±0.14 [0.71,0.94]	0.82±0.11 [0.72,0.92]	0.72±0.16 [0.58,0.86]	0.95±0.08	0.81±0.15	0.34±0.28	0.99±0.01	0.69±0.34	0.49±0.35
Patch	CONCH	default	0.09±0.05 [0.04,0.14]	0.42±0.11 [0.33,0.51]	64	0.3	(128, 64)	0.72±0.19 [0.55,0.89]	0.82±0.13 [0.71,0.93]	0.84±0.10 [0.74,0.93]	0.76±0.15 [0.63,0.89]	0.94±0.11	0.86±0.12	0.45±0.26	0.99±0.01	0.63±0.33	0.65±0.23
Patch	ResNet50	stainnorm	0.09±0.03 [0.06,0.12]	0.45±0.04 [0.41,0.49]	128	0.5	(128, 64)	0.72±0.19 [0.55,0.88]	0.82±0.12 [0.72,0.93]	0.84±0.11 [0.75,0.93]	0.76±0.15 [0.63,0.89]	0.94±0.08	0.87±0.11	0.40±0.20	0.99±0.01	0.64±0.30	0.65±0.27
Patch	ResNet50	stainnorm	0.09±0.03 [0.07,0.12]	0.46±0.05 [0.42,0.51]	64	0.3	(64, 32)	0.72±0.18 [0.56,0.88]	0.81±0.13 [0.70,0.93]	0.84±0.10 [0.76,0.93]	0.77±0.14 [0.64,0.89]	0.95±0.08	0.89±0.13	0.42±0.26	0.99±0.02	0.60±0.32	0.71±0.26
Patch	CONCH	stainnorm	0.11±0.03 [0.08,0.13]	0.43±0.04 [0.40,0.46]	64	0.3	(32, 16)	0.72±0.16 [0.58,0.87]	0.82±0.11 [0.72,0.92]	0.85±0.08 [0.78,0.92]	0.77±0.11 [0.68,0.87]	0.96±0.06	0.88±0.06	0.40±0.21	**1.00±0.00**	0.61±0.29	0.71±0.08

Patch	CONCH	default	0.05±0.02 [0.03,0.07]	0.38±0.09 [0.30,0.46]	128	0.5	(128, 64)	0.71±0.20 [0.53,0.88]	0.81±0.13 [0.69,0.92]	0.84±0.10 [0.75,0.93]	0.77±0.14 [0.65,0.89]	0.93±0.11	0.90±0.12	0.46±0.32	**1.00±0.00**	0.57±0.34	0.73±0.14
Patch	UNI	gray	0.07±0.05 [0.02,0.12]	0.38±0.11 [0.29,0.48]	64	0.3	(32, 16)	0.71±0.20 [0.53,0.88]	0.81±0.14 [0.69,0.93]	0.82±0.11 [0.72,0.92]	0.73±0.17 [0.58,0.88]	0.94±0.07	0.85±0.20	0.41±0.23	**1.00±0.00**	0.61±0.35	0.58±0.35
Patch	CONCH	stainnorm	0.08±0.04 [0.04,0.11]	0.45±0.03 [0.43,0.48]	64	0.3	(64, 32)	0.71±0.19 [0.55,0.88]	0.81±0.13 [0.70,0.92]	0.85±0.09 [0.77,0.93]	0.79±0.11 [0.69,0.89]	0.93±0.10	0.90±0.11	0.42±0.19	**1.00±0.00**	0.56±0.34	0.80±0.15
Patch	UNI	gray	0.05±0.05 [0.00,0.09]	0.43±0.11 [0.34,0.53]	64	0.5	(128, 64)	0.71±0.14 [0.58,0.83]	0.80±0.12 [0.69,0.90]	0.84±0.07 [0.78,0.90]	0.76±0.09 [0.68,0.84]	0.97±0.05	0.88±0.13	0.36±0.17	**1.00±0.00**	0.54±0.33	0.74±0.19
Patch	CONCH	stainnorm	0.09±0.05 [0.05,0.13]	0.44±0.03 [0.41,0.46]	128	0.3	(64, 32)	0.70±0.22 [0.51,0.90]	0.81±0.14 [0.68,0.93]	0.83±0.12 [0.73,0.94]	0.76±0.17 [0.61,0.90]	0.94±0.10	0.85±0.17	0.42±0.27	**1.00±0.00**	0.57±0.35	0.70±0.23
Patch	CONCH	default	0.09±0.06 [0.03,0.14]	0.39±0.07 [0.33,0.45]	64	0.5	(32, 16)	0.70±0.19 [0.54,0.87]	0.82±0.12 [0.71,0.92]	0.82±0.10 [0.74,0.91]	0.74±0.13 [0.62,0.85]	0.93±0.12	0.89±0.09	0.43±0.33	**1.00±0.00**	0.62±0.29	0.59±0.20
Patch	ResNet50	stainnorm	0.07±0.02 [0.06,0.09]	0.44±0.04 [0.40,0.48]	64	0.3	(128, 64)	0.70±0.19 [0.53,0.87]	0.80±0.13 [0.69,0.92]	0.83±0.11 [0.74,0.93]	0.76±0.15 [0.62,0.89]	0.95±0.07	0.87±0.11	0.38±0.22	0.99±0.01	0.58±0.31	0.70±0.23
Patch	ResNet50	stainnorm	0.09±0.04 [0.06,0.12]	0.46±0.05 [0.41,0.50]	64	0.5	(128, 64)	0.70±0.18 [0.54,0.87]	0.81±0.12 [0.70,0.92]	0.83±0.11 [0.74,0.93]	0.75±0.16 [0.61,0.89]	0.95±0.06	0.86±0.13	0.37±0.22	0.99±0.01	0.61±0.30	0.65±0.27
Patch	ResNet50	stainnorm	0.09±0.04 [0.06,0.12]	0.47±0.06 [0.42,0.52]	128	0.5	(64, 32)	0.70±0.17 [0.55,0.85]	0.81±0.11 [0.71,0.91]	0.83±0.10 [0.74,0.92]	0.75±0.15 [0.62,0.88]	0.95±0.07	0.86±0.14	0.36±0.18	0.99±0.01	0.61±0.26	0.65±0.27
Patch	UNI	gray	0.06±0.04 [0.02,0.09]	0.40±0.08 [0.33,0.47]	128	0.3	(32, 16)	0.70±0.16 [0.56,0.84]	0.79±0.13 [0.68,0.91]	0.83±0.08 [0.76,0.91]	0.75±0.13 [0.64,0.86]	0.97±0.07	0.85±0.20	0.36±0.18	**1.00±0.00**	0.54±0.34	0.71±0.22
Patch	CONCH	stainnorm	0.09±0.07 [0.02,0.16]	0.49±0.06 [0.44,0.54]	64	0.5	(128, 64)	0.70±0.16 [0.56,0.84]	0.80±0.11 [0.71,0.90]	0.83±0.09 [0.76,0.91]	0.75±0.12 [0.64,0.86]	0.95±0.06	0.86±0.10	0.36±0.18	**1.00±0.00**	0.57±0.27	0.68±0.12
Patch	UNI	default	0.03±0.02 [0.01,0.05]	0.34±0.11 [0.25,0.44]	128	0.3	(64, 32)	0.70±0.16 [0.56,0.84]	0.80±0.10 [0.72,0.89]	0.83±0.10 [0.75,0.92]	0.76±0.15 [0.63,0.89]	0.93±0.09	0.90±0.13	0.37±0.20	**1.00±0.00**	0.56±0.24	0.72±0.32
Patch	CONCH	default	0.04±0.03 [0.02,0.07]	0.37±0.09 [0.29,0.45]	64	0.3	(64, 32)	0.69±0.19 [0.52,0.86]	0.81±0.12 [0.71,0.91]	0.82±0.11 [0.73,0.92]	0.74±0.15 [0.61,0.87]	0.91±0.13	0.90±0.10	0.41±0.25	**1.00±0.00**	0.60±0.28	0.62±0.19
Patch	ResNet50	stainnorm	0.13±0.10 [0.04,0.22]	0.45±0.05 [0.41,0.50]	128	0.5	(32, 16)	0.69±0.18 [0.54,0.85]	0.81±0.12 [0.71,0.92]	0.81±0.12 [0.71,0.91]	0.71±0.17 [0.56,0.86]	0.94±0.08	0.81±0.15	0.27±0.22	0.99±0.01	0.66±0.26	0.48±0.36
Patch	ResNet50	stainnorm	0.10±0.04 [0.06,0.13]	0.47±0.07 [0.41,0.54]	128	0.3	(64, 32)	0.69±0.18 [0.53,0.85]	0.80±0.12 [0.70,0.91]	0.83±0.10 [0.74,0.92]	0.75±0.15 [0.61,0.88]	0.94±0.09	0.86±0.12	0.37±0.24	0.98±0.03	0.61±0.28	0.65±0.27
Patch	CONCH	stainnorm	0.15±0.08 [0.08,0.22]	0.45±0.05 [0.41,0.49]	64	0.5	(32, 16)	0.69±0.17 [0.54,0.84]	0.80±0.12 [0.70,0.91]	0.82±0.08 [0.75,0.89]	0.73±0.11 [0.63,0.82]	0.95±0.08	0.83±0.12	0.35±0.15	**1.00±0.00**	0.59±0.34	0.60±0.24
Patch	UNI	gray	0.06±0.05 [0.02,0.10]	0.47±0.11 [0.37,0.56]	64	0.3	(128, 64)	0.69±0.14 [0.57,0.81]	0.79±0.11 [0.70,0.88]	0.83±0.08 [0.76,0.89]	0.74±0.12 [0.63,0.85]	0.97±0.07	0.87±0.14	0.34±0.18	**1.00±0.00**	0.54±0.29	0.68±0.26
Patch	UNI	default	0.02±0.02 [0.00,0.04]	0.35±0.13 [0.24,0.46]	64	0.5	(128, 64)	0.69±0.11 [0.59,0.79]	0.80±0.07 [0.74,0.86]	0.84±0.07 [0.77,0.90]	0.77±0.11 [0.67,0.86]	0.92±0.09	0.91±0.10	0.35±0.13	**1.00±0.00**	0.55±0.16	0.75±0.25
Patch	CONCH	default	0.06±0.04 [0.03,0.09]	0.35±0.06 [0.30,0.40]	64	0.5	(64, 32)	0.68±0.21 [0.50,0.86]	0.79±0.13 [0.68,0.91]	0.82±0.11 [0.72,0.92]	0.74±0.15 [0.61,0.87]	0.92±0.12	0.87±0.14	0.43±0.33	**1.00±0.00**	0.55±0.32	0.67±0.21

Patch	UNI	gray	0.08±0.06 [0.03,0.14]	0.40±0.08 [0.32,0.47]	128	0.5	(32, 16)	0.68±0.18 [0.52,0.84]	0.79±0.13 [0.67,0.90]	0.83±0.09 [0.74,0.91]	0.75±0.13 [0.63,0.86]	0.94±0.07	0.84±0.25	0.35±0.16	**1.00±0.00**	0.51±0.34	0.73±0.20
Patch	CONCH	stainnorm	0.08±0.04 [0.04,0.12]	0.40±0.03 [0.37,0.42]	128	0.5	(32, 16)	0.68±0.17 [0.53,0.82]	0.79±0.11 [0.69,0.89]	0.82±0.08 [0.75,0.89]	0.74±0.10 [0.65,0.82]	0.93±0.09	0.88±0.08	0.35±0.16	0.99±0.02	0.55±0.29	0.68±0.16
Patch	ResNet50	stainnorm	0.10±0.03 [0.08,0.13]	0.46±0.06 [0.41,0.51]	64	0.3	(32, 16)	0.68±0.15 [0.55,0.81]	0.80±0.10 [0.71,0.89]	0.81±0.09 [0.74,0.89]	0.72±0.13 [0.60,0.83]	0.94±0.08	0.86±0.12	0.29±0.15	0.99±0.01	0.60±0.26	0.57±0.33
Patch	UNI	gray	0.07±0.05 [0.03,0.11]	0.42±0.09 [0.33,0.50]	64	0.5	(64, 32)	0.68±0.14 [0.56,0.80]	0.79±0.11 [0.69,0.89]	0.82±0.08 [0.75,0.89]	0.74±0.12 [0.63,0.84]	0.94±0.05	0.84±0.19	0.31±0.14	**1.00±0.00**	0.53±0.29	0.68±0.23
Patch	CONCH	default	0.06±0.04 [0.03,0.09]	0.36±0.10 [0.27,0.44]	128	0.3	(32, 16)	0.67±0.25 [0.46,0.89]	0.79±0.16 [0.65,0.93]	0.81±0.13 [0.70,0.92]	0.73±0.17 [0.58,0.88]	0.91±0.14	0.86±0.10	0.45±0.34	0.98±0.03	0.57±0.36	0.63±0.16
Patch	CONCH	default	0.06±0.03 [0.04,0.09]	0.37±0.08 [0.30,0.45]	128	0.5	(64, 32)	0.67±0.21 [0.48,0.86]	0.79±0.13 [0.68,0.91]	0.81±0.12 [0.71,0.91]	0.72±0.16 [0.58,0.86]	0.91±0.13	0.86±0.10	0.39±0.26	0.99±0.01	0.57±0.33	0.60±0.17
Patch	CONCH	default	0.06±0.07 [-0.00,0.12]	0.37±0.10 [0.28,0.46]	128	0.3	(64, 32)	0.67±0.20 [0.49,0.85]	0.79±0.12 [0.68,0.90]	0.81±0.11 [0.72,0.91]	0.73±0.15 [0.59,0.86]	0.91±0.13	0.89±0.11	0.39±0.26	**1.00±0.00**	0.55±0.29	0.64±0.20
Patch	CONCH	gray	0.09±0.07 [0.03,0.15]	0.45±0.06 [0.40,0.51]	64	0.5	(128, 64)	0.67±0.20 [0.49,0.85]	0.79±0.13 [0.68,0.90]	0.82±0.11 [0.72,0.92]	0.73±0.16 [0.60,0.87]	0.93±0.09	0.83±0.16	0.38±0.27	0.99±0.01	0.54±0.31	0.67±0.19
Patch	UNI	default	0.02±0.02 [0.00,0.04]	0.31±0.10 [0.22,0.39]	64	0.3	(32, 16)	0.67±0.19 [0.51,0.84]	0.79±0.12 [0.69,0.90]	0.82±0.11 [0.72,0.92]	0.75±0.17 [0.60,0.90]	0.91±0.10	0.86±0.21	0.38±0.20	**1.00±0.00**	0.53±0.27	0.72±0.32
Patch	ResNet50	stainnorm	0.07±0.02 [0.05,0.09]	0.41±0.03 [0.39,0.44]	128	0.3	(128, 64)	0.67±0.15 [0.53,0.80]	0.79±0.10 [0.70,0.87]	0.82±0.09 [0.73,0.90]	0.73±0.14 [0.61,0.85]	0.94±0.08	0.85±0.12	0.30±0.13	0.98±0.03	0.56±0.26	0.65±0.27
Patch	CONCH	default	0.06±0.03 [0.04,0.08]	0.37±0.07 [0.30,0.43]	64	0.5	(128, 64)	0.66±0.22 [0.47,0.85]	0.78±0.14 [0.66,0.91]	0.80±0.13 [0.69,0.91]	0.70±0.17 [0.55,0.86]	0.93±0.13	0.82±0.13	0.36±0.28	**1.00±0.00**	0.55±0.33	0.57±0.21
Patch	ResNet50	default	0.03±0.05 [-0.01,0.08]	0.34±0.15 [0.21,0.47]	128	0.5	(32, 16)	0.66±0.20 [0.48,0.83]	0.78±0.12 [0.67,0.89]	0.79±0.11 [0.69,0.89]	0.69±0.17 [0.53,0.84]	0.92±0.12	0.82±0.19	0.24±0.22	**1.00±0.00**	0.54±0.32	0.52±0.47
Patch	CONCH	gray	0.11±0.11 [0.01,0.20]	0.42±0.07 [0.36,0.49]	128	0.5	(128, 64)	0.66±0.19 [0.50,0.82]	0.77±0.13 [0.66,0.89]	0.81±0.09 [0.73,0.89]	0.73±0.12 [0.62,0.84]	0.93±0.10	0.69±0.39	0.35±0.21	0.99±0.01	0.51±0.35	0.69±0.14
Patch	CONCH	gray	0.10±0.07 [0.03,0.16]	0.43±0.06 [0.37,0.49]	64	0.3	(64, 32)	0.66±0.18 [0.50,0.82]	0.77±0.14 [0.65,0.89]	0.81±0.09 [0.73,0.89]	0.72±0.12 [0.62,0.82]	0.93±0.08	0.70±0.40	0.32±0.17	0.99±0.01	0.51±0.38	0.66±0.23
Patch	UNI	default	0.03±0.03 [0.01,0.05]	0.35±0.09 [0.27,0.42]	64	0.5	(64, 32)	0.66±0.18 [0.50,0.82]	0.78±0.12 [0.67,0.89]	0.80±0.11 [0.71,0.90]	0.71±0.17 [0.55,0.86]	0.93±0.07	0.84±0.15	0.37±0.31	**1.00±0.00**	0.53±0.28	0.59±0.34
Patch	CONCH	default	0.05±0.03 [0.02,0.08]	0.38±0.09 [0.30,0.46]	128	0.3	(128, 64)	0.66±0.17 [0.52,0.81]	0.79±0.11 [0.70,0.88]	0.81±0.09 [0.73,0.89]	0.72±0.12 [0.62,0.83]	0.91±0.12	0.88±0.11	0.35±0.17	**1.00±0.00**	0.55±0.27	0.62±0.15
Patch	ResNet50	default	0.01±0.01 [0.01,0.02]	0.37±0.11 [0.27,0.47]	128	0.3	(32, 16)	0.65±0.24 [0.44,0.86]	0.77±0.16 [0.63,0.91]	0.80±0.14 [0.68,0.93]	0.71±0.22 [0.52,0.90]	0.92±0.12	0.76±0.43	0.38±0.23	0.99±0.01	0.50±0.38	0.65±0.41

Patch	CONCH	gray	0.10±0.06 [0.05,0.15]	0.42±0.05 [0.38,0.47]	128	0.3	(32, 16)	0.65±0.22 [0.46,0.84]	0.77±0.15 [0.64,0.90]	0.80±0.10 [0.71,0.89]	0.70±0.14 [0.58,0.82]	0.92±0.10	0.72±0.41	0.38±0.35	**1.00±0.00**	0.51±0.40	0.59±0.27
Patch	CONCH	gray	0.05±0.03 [0.02,0.07]	0.42±0.03 [0.40,0.45]	128	0.3	(128, 64)	0.65±0.22 [0.46,0.83]	0.76±0.15 [0.63,0.89]	0.80±0.12 [0.70,0.91]	0.71±0.17 [0.56,0.86]	0.93±0.08	0.78±0.33	0.35±0.28	0.99±0.01	0.47±0.37	0.68±0.26
Patch	CONCH	gray	0.05±0.02 [0.03,0.07]	0.41±0.03 [0.38,0.44]	64	0.3	(32, 16)	0.65±0.19 [0.48,0.82]	0.77±0.13 [0.66,0.88]	0.80±0.11 [0.71,0.90]	0.71±0.15 [0.57,0.84]	0.93±0.08	0.82±0.23	0.31±0.19	0.99±0.01	0.50±0.34	0.62±0.29
Patch	UNI	default	0.04±0.03 [0.01,0.07]	0.39±0.17 [0.24,0.54]	64	0.3	(64, 32)	0.65±0.18 [0.50,0.80]	0.76±0.13 [0.64,0.87]	0.81±0.09 [0.73,0.89]	0.73±0.13 [0.62,0.84]	0.91±0.12	0.70±0.41	0.38±0.21	**1.00±0.00**	0.44±0.35	0.76±0.29
Patch	UNI	default	0.03±0.03 [0.00,0.05]	0.31±0.09 [0.23,0.38]	64	0.5	(32, 16)	0.65±0.17 [0.50,0.80]	0.76±0.13 [0.64,0.88]	0.81±0.10 [0.72,0.90]	0.73±0.15 [0.59,0.86]	0.91±0.09	0.70±0.41	0.36±0.22	**1.00±0.00**	0.45±0.34	0.72±0.35
Patch	CONCH	stainnorm	0.10±0.06 [0.05,0.15]	0.43±0.05 [0.38,0.47]	128	0.3	(32, 16)	0.65±0.13 [0.53,0.77]	0.77±0.09 [0.69,0.85]	0.81±0.07 [0.75,0.87]	0.72±0.10 [0.64,0.81]	0.93±0.08	0.86±0.09	0.30±0.13	0.99±0.01	0.50±0.23	0.67±0.14
Patch	ResNet50	default	0.02±0.01 [0.00,0.03]	0.40±0.16 [0.27,0.54]	128	0.3	(64, 32)	0.64±0.22 [0.45,0.83]	0.76±0.14 [0.64,0.89]	0.80±0.13 [0.69,0.92]	0.72±0.19 [0.55,0.88]	0.92±0.12	0.74±0.42	0.35±0.19	0.99±0.01	0.47±0.34	0.68±0.30
Patch	ResNet50	default	0.03±0.02 [0.01,0.04]	0.38±0.13 [0.26,0.49]	64	0.5	(128, 64)	0.64±0.20 [0.47,0.82]	0.77±0.13 [0.66,0.89]	0.78±0.11 [0.68,0.88]	0.67±0.18 [0.52,0.83]	0.92±0.12	0.80±0.20	0.24±0.22	0.99±0.01	0.54±0.34	0.49±0.45
Patch	CONCH	gray	0.12±0.08 [0.05,0.19]	0.44±0.07 [0.38,0.50]	64	0.5	(64, 32)	0.64±0.19 [0.48,0.81]	0.78±0.11 [0.68,0.88]	0.79±0.11 [0.69,0.88]	0.68±0.16 [0.54,0.82]	0.91±0.11	0.85±0.15	0.29±0.24	0.99±0.01	0.56±0.31	0.49±0.38
Patch	CONCH	gray	0.14±0.08 [0.06,0.21]	0.45±0.07 [0.38,0.51]	128	0.3	(64, 32)	0.64±0.17 [0.49,0.79]	0.77±0.10 [0.69,0.86]	0.77±0.08 [0.70,0.84]	0.65±0.12 [0.55,0.76]	0.91±0.11	0.83±0.14	0.17±0.16	**1.00±0.00**	0.55±0.30	0.41±0.43
Patch	UNI	default	0.01±0.02 [0.00,0.03]	0.38±0.17 [0.22,0.53]	64	0.3	(128, 64)	0.64±0.16 [0.51,0.78]	0.75±0.12 [0.64,0.86]	0.80±0.10 [0.72,0.89]	0.72±0.15 [0.59,0.85]	0.93±0.10	0.69±0.41	0.32±0.20	**1.00±0.00**	0.43±0.31	0.72±0.35
Patch	UNI	default	0.01±0.02 [0.00,0.03]	0.35±0.14 [0.23,0.47]	128	0.3	(128, 64)	0.64±0.10 [0.55,0.73]	0.74±0.10 [0.66,0.83]	0.80±0.07 [0.74,0.87]	0.72±0.12 [0.61,0.82]	0.95±0.10	0.71±0.41	0.28±0.17	**1.00±0.00**	0.40±0.26	0.75±0.34
Patch	ResNet50	default	0.02±0.03 [0.00,0.05]	0.36±0.16 [0.21,0.50]	128	0.3	(128, 64)	0.63±0.27 [0.40,0.87]	0.78±0.16 [0.63,0.92]	0.79±0.15 [0.66,0.92]	0.70±0.21 [0.51,0.88]	0.89±0.14	0.72±0.41	0.36±0.20	0.99±0.02	0.53±0.40	0.57±0.36
Patch	ResNet50	gray	0.03±0.01 [0.02,0.04]	0.40±0.13 [0.28,0.51]	128	0.3	(64, 32)	0.63±0.22 [0.43,0.82]	0.76±0.13 [0.64,0.88]	0.80±0.10 [0.71,0.89]	0.73±0.12 [0.63,0.83]	0.87±0.18	0.72±0.41	0.41±0.13	0.99±0.01	0.45±0.36	0.76±0.10
Patch	UNI	default	0.06±0.04 [0.02,0.09]	0.31±0.09 [0.23,0.39]	128	0.5	(32, 16)	0.63±0.21 [0.45,0.81]	0.77±0.14 [0.65,0.89]	0.78±0.11 [0.68,0.87]	0.66±0.15 [0.53,0.80]	0.92±0.11	0.73±0.23	0.35±0.30	**1.00±0.00**	0.53±0.34	0.46±0.21
Patch	ResNet50	default	0.02±0.01 [0.01,0.02]	0.40±0.18 [0.24,0.56]	64	0.3	(64, 32)	0.63±0.20 [0.45,0.80]	0.76±0.12 [0.65,0.87]	0.78±0.12 [0.67,0.89]	0.68±0.19 [0.51,0.85]	0.92±0.12	0.82±0.25	0.25±0.23	0.99±0.01	0.49±0.30	0.55±0.50
Patch	CONCH	gray	0.08±0.06 [0.03,0.14]	0.43±0.05 [0.39,0.47]	128	0.5	(64, 32)	0.63±0.18 [0.47,0.79]	0.76±0.12 [0.66,0.87]	0.79±0.10 [0.70,0.87]	0.68±0.15 [0.55,0.81]	0.93±0.07	0.81±0.18	0.26±0.18	0.99±0.01	0.51±0.31	0.54±0.28

Patch	UNI	default	0.02±0.02 [0.00,0.04]	0.33±0.08 [0.27,0.40]	128	0.5	(64, 32)	0.63±0.13 [0.52,0.74]	0.74±0.10 [0.65,0.83]	0.80±0.09 [0.72,0.87]	0.71±0.13 [0.59,0.82]	0.94±0.08	0.70±0.41	0.27±0.13	**1.00±0.00**	0.40±0.27	0.72±0.35
Patch	UNI	default	0.05±0.06 [0.00,0.10]	0.34±0.10 [0.25,0.43]	128	0.3	(32, 16)	0.63±0.12 [0.52,0.74]	0.75±0.10 [0.66,0.84]	0.80±0.08 [0.72,0.87]	0.71±0.12 [0.60,0.82]	0.90±0.11	0.69±0.40	0.33±0.21	**1.00±0.00**	0.42±0.27	0.71±0.33
Patch	ResNet50	default	0.01±0.01 [0.00,0.02]	0.33±0.14 [0.21,0.45]	64	0.5	(32, 16)	0.62±0.22 [0.43,0.81]	0.73±0.16 [0.59,0.87]	0.79±0.12 [0.68,0.89]	0.69±0.18 [0.54,0.85]	0.92±0.12	0.60±0.48	0.35±0.22	0.99±0.01	0.38±0.42	0.70±0.36
Patch	ResNet50	default	0.02±0.02 [0.00,0.04]	0.36±0.13 [0.25,0.48]	64	0.3	(32, 16)	0.62±0.19 [0.46,0.79]	0.74±0.14 [0.62,0.86]	0.79±0.12 [0.68,0.89]	0.69±0.17 [0.54,0.84]	0.92±0.11	0.65±0.40	0.32±0.22	0.99±0.02	0.43±0.36	0.65±0.39
Patch	CONCH	default	0.04±0.02 [0.02,0.05]	0.36±0.05 [0.32,0.40]	128	0.5	(32, 16)	0.62±0.18 [0.46,0.78]	0.75±0.12 [0.65,0.86]	0.79±0.10 [0.71,0.88]	0.70±0.13 [0.59,0.82]	0.91±0.13	0.88±0.13	0.29±0.12	0.98±0.05	0.48±0.26	0.65±0.13
Patch	CONCH	gray	0.11±0.08 [0.04,0.18]	0.45±0.05 [0.40,0.49]	64	0.3	(128, 64)	0.62±0.15 [0.49,0.76]	0.76±0.10 [0.67,0.85]	0.77±0.06 [0.72,0.82]	0.66±0.07 [0.59,0.72]	0.93±0.09	0.77±0.15	0.19±0.12	0.99±0.02	0.52±0.34	0.46±0.27
Patch	ResNet50	default	0.01±0.01 [0.01,0.02]	0.43±0.19 [0.26,0.60]	64	0.3	(128, 64)	0.61±0.26 [0.38,0.84]	0.75±0.17 [0.60,0.89]	0.78±0.16 [0.64,0.91]	0.67±0.23 [0.47,0.87]	0.91±0.14	0.74±0.41	0.33±0.24	0.99±0.01	0.46±0.36	0.57±0.36
Patch	CONCH	default	0.05±0.05 [0.00,0.09]	0.33±0.10 [0.25,0.42]	64	0.3	(32, 16)	0.61±0.14 [0.49,0.72]	0.74±0.09 [0.67,0.82]	0.78±0.08 [0.71,0.85]	0.68±0.11 [0.58,0.77]	0.91±0.12	0.84±0.17	0.26±0.09	**1.00±0.00**	0.44±0.22	0.59±0.19
Patch	CONCH	gray	0.04±0.02 [0.02,0.06]	0.41±0.03 [0.39,0.44]	128	0.5	(32, 16)	0.61±0.10 [0.53,0.70]	0.73±0.08 [0.66,0.80]	0.79±0.05 [0.75,0.83]	0.71±0.06 [0.66,0.76]	0.93±0.08	0.83±0.20	0.25±0.04	**1.00±0.00**	0.37±0.24	0.75±0.21
Patch	ResNet50	gray	0.05±0.06 [-0.01,0.10]	0.39±0.10 [0.30,0.47]	64	0.5	(128, 64)	0.60±0.21 [0.41,0.78]	0.73±0.13 [0.62,0.85]	0.77±0.09 [0.68,0.85]	0.66±0.13 [0.55,0.78]	0.89±0.15	0.72±0.41	0.24±0.20	**1.00±0.00**	0.41±0.37	0.58±0.41
Patch	ResNet50	gray	0.05±0.04 [0.01,0.08]	0.40±0.12 [0.29,0.50]	64	0.3	(128, 64)	0.60±0.20 [0.43,0.78]	0.74±0.12 [0.64,0.85]	0.77±0.08 [0.70,0.85]	0.68±0.11 [0.58,0.78]	0.88±0.16	0.73±0.41	0.26±0.19	**1.00±0.00**	0.42±0.36	0.62±0.39
Patch	ResNet50	gray	0.05±0.07 [-0.00,0.11]	0.41±0.16 [0.27,0.55]	64	0.3	(64, 32)	0.60±0.20 [0.42,0.77]	0.74±0.12 [0.63,0.85]	0.77±0.08 [0.69,0.84]	0.67±0.10 [0.58,0.76]	0.88±0.16	0.70±0.39	0.24±0.17	0.99±0.01	0.42±0.38	0.59±0.36
Patch	CONCH	gray	0.09±0.06 [0.03,0.14]	0.44±0.04 [0.40,0.47]	64	0.5	(32, 16)	0.60±0.11 [0.50,0.69]	0.73±0.08 [0.66,0.80]	0.78±0.05 [0.73,0.82]	0.67±0.06 [0.62,0.72]	0.94±0.07	0.79±0.12	0.22±0.04	0.99±0.02	0.42±0.26	0.60±0.21
Patch	ResNet50	gray	0.04±0.04 [0.00,0.07]	0.42±0.17 [0.27,0.57]	128	0.3	(128, 64)	0.59±0.22 [0.40,0.78]	0.73±0.13 [0.62,0.84]	0.76±0.08 [0.69,0.84]	0.67±0.10 [0.58,0.75]	0.87±0.18	0.72±0.41	0.26±0.19	**1.00±0.00**	0.39±0.38	0.62±0.39
Patch	ResNet50	gray	0.04±0.03 [0.02,0.07]	0.37±0.12 [0.27,0.47]	128	0.5	(128, 64)	0.58±0.26 [0.35,0.81]	0.73±0.14 [0.61,0.86]	0.75±0.13 [0.64,0.86]	0.64±0.18 [0.48,0.79]	0.88±0.17	0.70±0.39	0.21±0.21	**1.00±0.00**	0.43±0.37	0.49±0.45
Patch	ResNet50	gray	0.09±0.07 [0.03,0.16]	0.38±0.12 [0.27,0.49]	128	0.3	(32, 16)	0.58±0.26 [0.35,0.81]	0.74±0.15 [0.61,0.87]	0.75±0.11 [0.65,0.84]	0.63±0.14 [0.51,0.75]	0.87±0.18	0.54±0.43	0.20±0.16	**1.00±0.00**	0.46±0.45	0.44±0.46
Patch	ResNet50	default	0.01±0.01 [0.00,0.02]	0.39±0.17 [0.24,0.54]	128	0.5	(128, 64)	0.58±0.25 [0.36,0.80]	0.73±0.15 [0.60,0.86]	0.76±0.13 [0.64,0.88]	0.65±0.19 [0.48,0.82]	0.89±0.14	0.54±0.50	0.20±0.18	0.99±0.01	0.41±0.41	0.55±0.50
Patch	ResNet50	default	0.02±0.02 [0.01,0.04]	0.40±0.19 [0.23,0.56]	64	0.5	(64, 32)	0.58±0.23 [0.38,0.78]	0.74±0.13 [0.62,0.86]	0.76±0.14 [0.64,0.88]	0.65±0.21 [0.47,0.83]	0.89±0.15	0.71±0.40	0.25±0.23	0.99±0.01	0.44±0.32	0.52±0.47

Patch	ResNet50	default	0.02±0.02 [0.00,0.03]	0.38±0.18 [0.22,0.54]	128	0.5	(64, 32)	0.58±0.23 [0.37,0.78]	0.71±0.15 [0.57,0.84]	0.75±0.12 [0.65,0.86]	0.64±0.17 [0.49,0.79]	0.90±0.13	0.53±0.49	0.30±0.21	**1.00±0.00**	0.34±0.40	0.58±0.46
Patch	ResNet50	gray	0.14±0.26 [-0.09,0.36]	0.45±0.13 [0.33,0.56]	64	0.5	(64, 32)	0.58±0.17 [0.43,0.73]	0.73±0.10 [0.64,0.82]	0.77±0.07 [0.70,0.83]	0.67±0.09 [0.60,0.75]	0.88±0.17	0.68±0.38	0.27±0.18	0.97±0.06	0.42±0.37	0.62±0.36
Patch	ResNet50	gray	0.05±0.05 [0.00,0.09]	0.35±0.06 [0.30,0.41]	128	0.5	(32, 16)	0.56±0.23 [0.36,0.76]	0.72±0.13 [0.60,0.83]	0.75±0.09 [0.67,0.82]	0.64±0.11 [0.54,0.73]	0.86±0.17	0.74±0.31	0.24±0.20	0.99±0.01	0.39±0.37	0.53±0.37
Patch	ResNet50	gray	0.12±0.23 [-0.08,0.32]	0.42±0.15 [0.29,0.55]	128	0.5	(64, 32)	0.56±0.21 [0.38,0.74]	0.71±0.12 [0.61,0.82]	0.75±0.08 [0.68,0.82]	0.65±0.10 [0.57,0.73]	0.87±0.18	0.50±0.46	0.23±0.15	0.98±0.04	0.37±0.41	0.59±0.39
Patch	ResNet50	gray	0.13±0.24 [-0.08,0.34]	0.42±0.12 [0.31,0.53]	64	0.3	(32, 16)	0.56±0.18 [0.40,0.71]	0.72±0.10 [0.63,0.80]	0.75±0.07 [0.68,0.81]	0.64±0.09 [0.56,0.72]	0.87±0.17	0.65±0.37	0.24±0.20	0.97±0.06	0.41±0.38	0.53±0.36
Patch	ResNet50	gray	0.03±0.04 [-0.00,0.06]	0.31±0.04 [0.27,0.35]	64	0.5	(32, 16)	0.55±0.22 [0.36,0.75]	0.71±0.13 [0.59,0.83]	0.75±0.09 [0.67,0.82]	0.65±0.10 [0.56,0.73]	0.85±0.16	0.52±0.48	0.23±0.14	0.99±0.01	0.36±0.40	0.59±0.42

Supplementary Table 3

Test results for all classifiers.

The metrics display the 5-Fold CV mean, along with its standard deviation and 95% confidence interval, based on the validation set. c1: lower Youden J threshold, c2: upper Youden J threshold, B: batch size, D: dropout rate, HS: hidden sizes.

Model	Encoder	Normalization	c1	c2	HS	D	B	HNS Accuracy	HNS F1	HNS Precision	HNS Recall
mCLAM	ResNet50	stainnorm	N/A	N/A	N/A	N/A	N/A	**0.96±0.05 [0.92,1.01]**	**0.98±0.03 [0.96,1.01]**	**1.00±0.00 [1.00,1.00]**	**0.96±0.05 [0.92,1.01]**
Patch	ResNet50	stainnorm	0.07±0.02 [0.06,0.09]	0.44±0.04 [0.40,0.48]	64	0.3	(128, 64)	0.79±0.03 [0.76,0.82]	0.88±0.02 [0.86,0.90]	**1.00±0.00 [1.00,1.00]**	0.79±0.03 [0.76,0.82]
mCLAM	ResNet50	default	N/A	N/A	N/A	N/A	N/A	0.76±0.25 [0.55,0.98]	0.85±0.19 [0.68,1.01]	**1.00±0.00 [1.00,1.00]**	0.76±0.25 [0.55,0.98]
mCLAM	ResNet50	gray	N/A	N/A	N/A	N/A	N/A	0.72±0.16 [0.57,0.86]	0.83±0.12 [0.72,0.93]	**1.00±0.00 [1.00,1.00]**	0.72±0.16 [0.57,0.86]
mCLAM	CONCH	gray	N/A	N/A	N/A	N/A	N/A	0.71±0.14 [0.58,0.83]	0.82±0.10 [0.73,0.91]	**1.00±0.00 [1.00,1.00]**	0.71±0.14 [0.58,0.83]
Patch	ResNet50	stainnorm	0.10±0.04 [0.06,0.13]	0.47±0.07 [0.41,0.54]	128	0.3	(64, 32)	0.71±0.12 [0.60,0.81]	0.82±0.08 [0.75,0.89]	**1.00±0.00 [1.00,1.00]**	0.71±0.12 [0.60,0.81]
Patch	ResNet50	stainnorm	0.10±0.03 [0.08,0.13]	0.46±0.06 [0.41,0.51]	64	0.3	(32, 16)	0.71±0.09 [0.62,0.79]	0.82±0.06 [0.77,0.88]	**1.00±0.00 [1.00,1.00]**	0.71±0.09 [0.62,0.79]
Patch	ResNet50	stainnorm	0.09±0.04 [0.06,0.12]	0.46±0.05 [0.41,0.50]	64	0.5	(128, 64)	0.69±0.06 [0.64,0.75]	0.82±0.04 [0.78,0.86]	**1.00±0.00 [1.00,1.00]**	0.69±0.06 [0.64,0.75]
Patch	ResNet50	stainnorm	0.09±0.03 [0.07,0.12]	0.46±0.05 [0.42,0.51]	64	0.3	(64, 32)	0.67±0.08 [0.60,0.74]	0.80±0.06 [0.75,0.85]	**1.00±0.00 [1.00,1.00]**	0.67±0.08 [0.60,0.74]

CLAM	ResNet50	stainnorm	N/A	N/A	N/A	N/A	N/A	0.73±0.35 [0.42,1.04]	0.79±0.33 [0.50,1.08]	**1.00±0.00 [1.00,1.00]**	0.73±0.35 [0.42,1.04]
Patch	ResNet50	stainnorm	0.09±0.04 [0.06,0.12]	0.47±0.06 [0.42,0.52]	128	0.5	(64, 32)	0.66±0.14 [0.54,0.78]	0.79±0.10 [0.70,0.87]	**1.00±0.00 [1.00,1.00]**	0.66±0.14 [0.54,0.78]
Patch	ResNet50	stainnorm	0.07±0.02 [0.05,0.09]	0.41±0.03 [0.39,0.44]	128	0.3	(128, 64)	0.66±0.10 [0.57,0.74]	0.79±0.07 [0.73,0.85]	**1.00±0.00 [1.00,1.00]**	0.66±0.10 [0.57,0.74]
Patch	ResNet50	stainnorm	0.09±0.03 [0.06,0.12]	0.45±0.04 [0.41,0.49]	128	0.5	(128, 64)	0.65±0.17 [0.50,0.80]	0.77±0.14 [0.65,0.90]	**1.00±0.00 [1.00,1.00]**	0.65±0.17 [0.50,0.80]
Patch	ResNet50	stainnorm	0.11±0.02 [0.09,0.13]	0.49±0.08 [0.42,0.56]	64	0.5	(64, 32)	0.64±0.09 [0.56,0.71]	0.77±0.06 [0.72,0.83]	**1.00±0.00 [1.00,1.00]**	0.64±0.09 [0.56,0.71]
Patch	ResNet50	stainnorm	0.14±0.11 [0.04,0.24]	0.46±0.06 [0.41,0.51]	64	0.5	(32, 16)	0.64±0.17 [0.48,0.79]	0.76±0.15 [0.64,0.89]	**1.00±0.00 [1.00,1.00]**	0.64±0.17 [0.48,0.79]
Patch	ResNet50	stainnorm	0.15±0.11 [0.05,0.25]	0.48±0.06 [0.43,0.53]	128	0.3	(32, 16)	0.65±0.29 [0.40,0.90]	0.75±0.23 [0.56,0.95]	**1.00±0.00 [1.00,1.00]**	0.65±0.29 [0.40,0.90]
Patch	UNI	gray	0.06±0.05 [0.02,0.10]	0.47±0.11 [0.37,0.56]	64	0.3	(128, 64)	0.62±0.22 [0.43,0.82]	0.75±0.18 [0.59,0.91]	**1.00±0.00 [1.00,1.00]**	0.62±0.22 [0.43,0.82]
Patch	ResNet50	stainnorm	0.13±0.10 [0.04,0.22]	0.45±0.05 [0.41,0.50]	128	0.5	(32, 16)	0.62±0.21 [0.44,0.80]	0.75±0.16 [0.61,0.89]	**1.00±0.00 [1.00,1.00]**	0.62±0.21 [0.44,0.80]
mCLAM	CONCH	stainnorm	N/A	N/A	N/A	N/A	N/A	0.64±0.29 [0.38,0.89]	0.74±0.24 [0.53,0.95]	**1.00±0.00 [1.00,1.00]**	0.64±0.29 [0.38,0.89]
Patch	UNI	gray	0.02±0.01 [0.02,0.03]	0.40±0.08 [0.33,0.47]	128	0.5	(128, 64)	0.60±0.29 [0.35,0.85]	0.72±0.24 [0.51,0.93]	**1.00±0.00 [1.00,1.00]**	0.60±0.29 [0.35,0.85]
CLAM	CONCH	stainnorm	N/A	N/A	N/A	N/A	N/A	0.59±0.22 [0.40,0.78]	0.72±0.16 [0.58,0.87]	**1.00±0.00 [1.00,1.00]**	0.59±0.22 [0.40,0.78]
CLAM	CONCH	gray	N/A	N/A	N/A	N/A	N/A	0.65±0.38 [0.31,0.98]	0.71±0.40 [0.36,1.07]	0.80±0.45 [0.41,1.19]	0.65±0.38 [0.31,0.98]
Patch	UNI	gray	0.04±0.03 [0.02,0.07]	0.41±0.07 [0.36,0.47]	128	0.3	(128, 64)	0.56±0.13 [0.45,0.68]	0.71±0.11 [0.61,0.82]	**1.00±0.00 [1.00,1.00]**	0.56±0.13 [0.45,0.68]
Patch	ResNet50	gray	0.05±0.04 [0.01,0.08]	0.40±0.12 [0.29,0.50]	64	0.3	(128, 64)	0.56±0.13 [0.45,0.68]	0.71±0.11 [0.61,0.82]	**1.00±0.00 [1.00,1.00]**	0.56±0.13 [0.45,0.68]
mMIL	CONCH	stainnorm	N/A	N/A	N/A	N/A	N/A	0.55±0.09 [0.48,0.63]	0.71±0.08 [0.64,0.77]	**1.00±0.00 [1.00,1.00]**	0.55±0.09 [0.48,0.63]
Patch	CONCH	stainnorm	0.08±0.02 [0.06,0.10]	0.45±0.02 [0.44,0.47]	64	0.5	(64, 32)	0.54±0.03 [0.52,0.56]	0.70±0.02 [0.68,0.72]	**1.00±0.00 [1.00,1.00]**	0.54±0.03 [0.52,0.56]
Patch	ResNet50	gray	0.05±0.06 [-0.01,0.10]	0.39±0.10 [0.30,0.47]	64	0.5	(128, 64)	0.59±0.34 [0.29,0.89]	0.69±0.28 [0.45,0.94]	**1.00±0.00 [1.00,1.00]**	0.59±0.34 [0.29,0.89]
Patch	UNI	gray	0.05±0.05 [0.00,0.09]	0.43±0.11 [0.34,0.53]	64	0.5	(128, 64)	0.51±0.10 [0.42,0.59]	0.67±0.09 [0.59,0.75]	**1.00±0.00 [1.00,1.00]**	0.51±0.10 [0.42,0.59]
Patch	CONCH	stainnorm	0.07±0.02 [0.05,0.09]	0.43±0.06 [0.38,0.48]	128	0.3	(128, 64)	0.48±0.06 [0.43,0.54]	0.65±0.06 [0.60,0.70]	**1.00±0.00 [1.00,1.00]**	0.48±0.06 [0.43,0.54]
Patch	ResNet50	gray	0.04±0.04 [0.00,0.07]	0.42±0.17 [0.27,0.57]	128	0.3	(128, 64)	0.49±0.19 [0.33,0.66]	0.64±0.18 [0.49,0.80]	**1.00±0.00 [1.00,1.00]**	0.49±0.19 [0.33,0.66]
Patch	UNI	stainnorm	0.03±0.01 [0.02,0.04]	0.39±0.04 [0.36,0.42]	64	0.3	(32, 16)	0.47±0.11 [0.37,0.57]	0.63±0.10 [0.55,0.72]	**1.00±0.00 [1.00,1.00]**	0.47±0.11 [0.37,0.57]
Patch	CONCH	stainnorm	0.07±0.02 [0.05,0.08]	0.45±0.05 [0.41,0.49]	128	0.5	(128, 64)	0.46±0.08 [0.39,0.53]	0.63±0.07 [0.56,0.69]	**1.00±0.00 [1.00,1.00]**	0.46±0.08 [0.39,0.53]

Patch	UNI	gray	0.07±0.05 [0.03,0.11]	0.42±0.09 [0.33,0.50]	64	0.5	(64, 32)	0.48±0.23 [0.28,0.69]	0.62±0.22 [0.43,0.81]	**1.00±0.00 [1.00,1.00]**	0.48±0.23 [0.28,0.69]
Patch	UNI	gray	0.09±0.06 [0.04,0.15]	0.40±0.07 [0.33,0.46]	64	0.5	(32, 16)	0.47±0.20 [0.30,0.65]	0.62±0.21 [0.44,0.80]	**1.00±0.00 [1.00,1.00]**	0.47±0.20 [0.30,0.65]
Patch	CONCH	stainnorm	0.08±0.04 [0.04,0.11]	0.45±0.03 [0.43,0.48]	64	0.3	(64, 32)	0.46±0.11 [0.36,0.56]	0.62±0.11 [0.53,0.72]	**1.00±0.00 [1.00,1.00]**	0.46±0.11 [0.36,0.56]
Patch	CONCH	gray	0.09±0.07 [0.03,0.15]	0.45±0.06 [0.40,0.51]	64	0.5	(128, 64)	0.45±0.13 [0.33,0.56]	0.61±0.12 [0.50,0.71]	**1.00±0.00 [1.00,1.00]**	0.45±0.13 [0.33,0.56]
Patch	UNI	gray	0.06±0.05 [0.02,0.11]	0.44±0.11 [0.34,0.53]	128	0.3	(64, 32)	0.45±0.12 [0.34,0.55]	0.61±0.11 [0.51,0.71]	**1.00±0.00 [1.00,1.00]**	0.45±0.12 [0.34,0.55]
Patch	CONCH	stainnorm	0.09±0.07 [0.02,0.16]	0.49±0.06 [0.44,0.54]	64	0.5	(128, 64)	0.45±0.08 [0.38,0.52]	0.61±0.08 [0.55,0.68]	**1.00±0.00 [1.00,1.00]**	0.45±0.08 [0.38,0.52]
Patch	CONCH	default	0.06±0.04 [0.03,0.09]	0.35±0.06 [0.30,0.40]	64	0.5	(64, 32)	0.44±0.05 [0.39,0.48]	0.61±0.05 [0.56,0.65]	**1.00±0.00 [1.00,1.00]**	0.44±0.05 [0.39,0.48]
Patch	UNI	stainnorm	0.04±0.01 [0.03,0.04]	0.42±0.03 [0.39,0.45]	128	0.3	(64, 32)	0.44±0.05 [0.39,0.48]	0.61±0.05 [0.56,0.65]	**1.00±0.00 [1.00,1.00]**	0.44±0.05 [0.39,0.48]
Patch	CONCH	gray	0.05±0.03 [0.02,0.07]	0.42±0.03 [0.40,0.45]	128	0.3	(128, 64)	0.44±0.05 [0.39,0.48]	0.61±0.05 [0.56,0.65]	**1.00±0.00 [1.00,1.00]**	0.44±0.05 [0.39,0.48]
Patch	UNI	stainnorm	0.04±0.02 [0.02,0.06]	0.41±0.06 [0.35,0.46]	64	0.3	(128, 64)	0.44±0.05 [0.39,0.48]	0.61±0.05 [0.56,0.65]	**1.00±0.00 [1.00,1.00]**	0.44±0.05 [0.39,0.48]
Patch	CONCH	default	0.06±0.04 [0.03,0.09]	0.36±0.10 [0.27,0.44]	128	0.3	(32, 16)	0.44±0.03 [0.41,0.46]	0.61±0.03 [0.58,0.63]	**1.00±0.00 [1.00,1.00]**	0.44±0.03 [0.41,0.46]
Patch	CONCH	stainnorm	0.09±0.05 [0.05,0.13]	0.44±0.03 [0.41,0.46]	128	0.3	(64, 32)	0.44±0.14 [0.31,0.56]	0.60±0.14 [0.47,0.72]	**1.00±0.00 [1.00,1.00]**	0.44±0.14 [0.31,0.56]
Patch	CONCH	stainnorm	0.08±0.04 [0.04,0.12]	0.40±0.03 [0.37,0.42]	128	0.5	(32, 16)	0.44±0.09 [0.36,0.51]	0.60±0.09 [0.52,0.68]	**1.00±0.00 [1.00,1.00]**	0.44±0.09 [0.36,0.51]
Patch	UNI	stainnorm	0.05±0.02 [0.03,0.07]	0.40±0.05 [0.36,0.45]	64	0.5	(64, 32)	0.44±0.08 [0.37,0.50]	0.60±0.08 [0.54,0.67]	**1.00±0.00 [1.00,1.00]**	0.44±0.08 [0.37,0.50]
Patch	CONCH	default	0.04±0.03 [0.02,0.07]	0.37±0.09 [0.29,0.45]	64	0.3	(64, 32)	0.44±0.08 [0.37,0.50]	0.60±0.08 [0.53,0.68]	**1.00±0.00 [1.00,1.00]**	0.44±0.08 [0.37,0.50]
Patch	ResNet50	gray	0.04±0.03 [0.02,0.07]	0.37±0.12 [0.27,0.47]	128	0.5	(128, 64)	0.44±0.17 [0.29,0.58]	0.59±0.18 [0.43,0.75]	**1.00±0.00 [1.00,1.00]**	0.44±0.17 [0.29,0.58]
Patch	UNI	stainnorm	0.06±0.04 [0.02,0.10]	0.42±0.07 [0.36,0.48]	128	0.3	(32, 16)	0.44±0.15 [0.31,0.56]	0.59±0.15 [0.47,0.72]	**1.00±0.00 [1.00,1.00]**	0.44±0.15 [0.31,0.56]
mMIL	CONCH	gray	N/A	N/A	N/A	N/A	N/A	0.42±0.10 [0.34,0.51]	0.59±0.10 [0.50,0.68]	**1.00±0.00 [1.00,1.00]**	0.42±0.10 [0.34,0.51]
Patch	CONCH	gray	0.09±0.06 [0.03,0.14]	0.44±0.04 [0.40,0.47]	64	0.5	(32, 16)	0.42±0.09 [0.35,0.50]	0.59±0.09 [0.51,0.67]	**1.00±0.00 [1.00,1.00]**	0.42±0.09 [0.35,0.50]
Patch	UNI	stainnorm	0.03±0.02 [0.01,0.04]	0.39±0.06 [0.33,0.45]	128	0.3	(128, 64)	0.42±0.08 [0.36,0.49]	0.59±0.08 [0.52,0.66]	**1.00±0.00 [1.00,1.00]**	0.42±0.08 [0.36,0.49]
Patch	CONCH	default	0.04±0.02 [0.02,0.05]	0.36±0.05 [0.32,0.40]	128	0.5	(32, 16)	0.42±0.08 [0.36,0.49]	0.59±0.08 [0.52,0.66]	**1.00±0.00 [1.00,1.00]**	0.42±0.08 [0.36,0.49]
Patch	UNI	stainnorm	0.04±0.02 [0.03,0.06]	0.37±0.04 [0.34,0.40]	64	0.5	(32, 16)	0.42±0.08 [0.36,0.49]	0.59±0.07 [0.53,0.66]	**1.00±0.00 [1.00,1.00]**	0.42±0.08 [0.36,0.49]

Patch	UNI	stainnorm	0.05±0.01 [0.03,0.06]	0.40±0.04 [0.37,0.44]	128	0.5	(64, 32)	0.42±0.06 [0.37,0.48]	0.59±0.06 [0.54,0.65]	**1.00±0.00 [1.00,1.00]**	0.42±0.06 [0.37,0.48]
Patch	UNI	gray	0.07±0.04 [0.04,0.11]	0.40±0.11 [0.30,0.50]	128	0.5	(64, 32)	0.46±0.32 [0.18,0.74]	0.58±0.29 [0.33,0.83]	**1.00±0.00 [1.00,1.00]**	0.46±0.32 [0.18,0.74]
Patch	UNI	gray	0.05±0.05 [0.01,0.09]	0.47±0.14 [0.35,0.59]	64	0.3	(64, 32)	0.44±0.23 [0.23,0.64]	0.58±0.25 [0.36,0.79]	**1.00±0.00 [1.00,1.00]**	0.44±0.23 [0.23,0.64]
Patch	CONCH	gray	0.11±0.08 [0.04,0.18]	0.45±0.05 [0.40,0.49]	64	0.3	(128, 64)	0.44±0.20 [0.26,0.61]	0.58±0.20 [0.41,0.76]	**1.00±0.00 [1.00,1.00]**	0.44±0.20 [0.26,0.61]
Patch	CONCH	gray	0.14±0.08 [0.06,0.21]	0.45±0.07 [0.38,0.51]	128	0.3	(64, 32)	0.42±0.16 [0.28,0.57]	0.58±0.14 [0.45,0.71]	**1.00±0.00 [1.00,1.00]**	0.42±0.16 [0.28,0.57]
Patch	CONCH	default	0.05±0.02 [0.03,0.07]	0.38±0.09 [0.30,0.46]	128	0.5	(128, 64)	0.41±0.11 [0.32,0.51]	0.58±0.12 [0.47,0.68]	**1.00±0.00 [1.00,1.00]**	0.41±0.11 [0.32,0.51]
Patch	CONCH	default	0.09±0.05 [0.04,0.14]	0.42±0.11 [0.33,0.51]	64	0.3	(128, 64)	0.41±0.12 [0.31,0.51]	0.58±0.11 [0.48,0.68]	**1.00±0.00 [1.00,1.00]**	0.41±0.12 [0.31,0.51]
Patch	CONCH	default	0.05±0.05 [0.00,0.09]	0.33±0.10 [0.25,0.42]	64	0.3	(32, 16)	0.41±0.07 [0.35,0.47]	0.58±0.08 [0.51,0.65]	**1.00±0.00 [1.00,1.00]**	0.41±0.07 [0.35,0.47]
Patch	UNI	stainnorm	0.05±0.04 [0.01,0.08]	0.40±0.04 [0.37,0.44]	64	0.3	(64, 32)	0.41±0.07 [0.35,0.47]	0.58±0.08 [0.51,0.65]	**1.00±0.00 [1.00,1.00]**	0.41±0.07 [0.35,0.47]
Patch	UNI	stainnorm	0.05±0.01 [0.05,0.06]	0.39±0.03 [0.37,0.42]	128	0.5	(32, 16)	0.41±0.04 [0.38,0.45]	0.58±0.04 [0.55,0.62]	**1.00±0.00 [1.00,1.00]**	0.41±0.04 [0.38,0.45]
Patch	ResNet50	gray	0.05±0.05 [0.00,0.09]	0.35±0.06 [0.30,0.41]	128	0.5	(32, 16)	0.51±0.44 [0.12,0.89]	0.57±0.44 [0.18,0.96]	0.80±0.45 [0.41,1.19]	0.51±0.44 [0.12,0.89]
Patch	UNI	gray	0.07±0.05 [0.02,0.12]	0.38±0.11 [0.29,0.48]	64	0.3	(32, 16)	0.45±0.31 [0.18,0.72]	0.57±0.27 [0.34,0.81]	**1.00±0.00 [1.00,1.00]**	0.45±0.31 [0.18,0.72]
Patch	ResNet50	gray	0.05±0.07 [-0.00,0.11]	0.41±0.16 [0.27,0.55]	64	0.3	(64, 32)	0.42±0.23 [0.22,0.63]	0.57±0.22 [0.37,0.76]	**1.00±0.00 [1.00,1.00]**	0.42±0.23 [0.22,0.63]
Patch	CONCH	default	0.09±0.06 [0.03,0.14]	0.39±0.07 [0.33,0.45]	64	0.5	(32, 16)	0.41±0.15 [0.28,0.54]	0.57±0.14 [0.45,0.70]	**1.00±0.00 [1.00,1.00]**	0.41±0.15 [0.28,0.54]
Patch	CONCH	default	0.06±0.03 [0.04,0.09]	0.37±0.08 [0.30,0.45]	128	0.5	(64, 32)	0.40±0.10 [0.32,0.48]	0.57±0.10 [0.48,0.65]	**1.00±0.00 [1.00,1.00]**	0.40±0.10 [0.32,0.48]
Patch	CONCH	default	0.06±0.07 [-0.00,0.12]	0.37±0.10 [0.28,0.46]	128	0.3	(64, 32)	0.40±0.10 [0.32,0.48]	0.57±0.10 [0.48,0.65]	**1.00±0.00 [1.00,1.00]**	0.40±0.10 [0.32,0.48]
Patch	CONCH	stainnorm	0.13±0.08 [0.05,0.20]	0.48±0.06 [0.42,0.53]	64	0.3	(128, 64)	0.40±0.09 [0.32,0.48]	0.57±0.09 [0.49,0.64]	**1.00±0.00 [1.00,1.00]**	0.40±0.09 [0.32,0.48]
Patch	UNI	stainnorm	0.05±0.02 [0.03,0.06]	0.41±0.06 [0.36,0.46]	64	0.5	(128, 64)	0.40±0.08 [0.33,0.47]	0.57±0.08 [0.50,0.64]	**1.00±0.00 [1.00,1.00]**	0.40±0.08 [0.33,0.47]
Patch	CONCH	gray	0.12±0.08 [0.05,0.19]	0.44±0.07 [0.38,0.50]	64	0.5	(64, 32)	0.40±0.06 [0.34,0.46]	0.57±0.07 [0.51,0.63]	**1.00±0.00 [1.00,1.00]**	0.40±0.06 [0.34,0.46]
Patch	UNI	stainnorm	0.04±0.02 [0.02,0.05]	0.38±0.04 [0.34,0.42]	128	0.5	(128, 64)	0.40±0.05 [0.36,0.44]	0.57±0.05 [0.53,0.61]	**1.00±0.00 [1.00,1.00]**	0.40±0.05 [0.36,0.44]
Patch	UNI	gray	0.08±0.06 [0.03,0.14]	0.40±0.08 [0.32,0.47]	128	0.5	(32, 16)	0.41±0.21 [0.23,0.59]	0.56±0.21 [0.38,0.74]	**1.00±0.00 [1.00,1.00]**	0.41±0.21 [0.23,0.59]
Patch	CONCH	gray	0.10±0.07 [0.03,0.16]	0.43±0.06 [0.37,0.49]	64	0.3	(64, 32)	0.40±0.13 [0.29,0.51]	0.56±0.14 [0.44,0.68]	**1.00±0.00 [1.00,1.00]**	0.40±0.13 [0.29,0.51]

Patch	CONCH	stainnorm	0.15±0.08 [0.08,0.22]	0.45±0.05 [0.41,0.49]	64	0.5	(32, 16)	0.40±0.11 [0.30,0.50]	0.56±0.12 [0.46,0.67]	**1.00±0.00 [1.00,1.00]**	0.40±0.11 [0.30,0.50]
Patch	CONCH	stainnorm	0.11±0.03 [0.08,0.13]	0.43±0.04 [0.40,0.46]	64	0.3	(32, 16)	0.40±0.11 [0.30,0.50]	0.56±0.12 [0.46,0.67]	**1.00±0.00 [1.00,1.00]**	0.40±0.11 [0.30,0.50]
Patch	CONCH	gray	0.05±0.02 [0.03,0.07]	0.41±0.03 [0.38,0.44]	64	0.3	(32, 16)	0.39±0.13 [0.28,0.50]	0.55±0.15 [0.42,0.68]	**1.00±0.00 [1.00,1.00]**	0.39±0.13 [0.28,0.50]
Patch	CONCH	default	0.05±0.03 [0.02,0.08]	0.38±0.09 [0.30,0.46]	128	0.3	(128, 64)	0.39±0.14 [0.27,0.51]	0.55±0.13 [0.43,0.67]	**1.00±0.00 [1.00,1.00]**	0.39±0.14 [0.27,0.51]
Patch	CONCH	stainnorm	0.14±0.06 [0.09,0.20]	0.45±0.03 [0.43,0.47]	128	0.5	(64, 32)	0.39±0.10 [0.30,0.47]	0.55±0.11 [0.46,0.65]	**1.00±0.00 [1.00,1.00]**	0.39±0.10 [0.30,0.47]
Patch	CONCH	gray	0.08±0.06 [0.03,0.14]	0.43±0.05 [0.39,0.47]	128	0.5	(64, 32)	0.39±0.11 [0.29,0.48]	0.55±0.11 [0.46,0.65]	**1.00±0.00 [1.00,1.00]**	0.39±0.11 [0.29,0.48]
CLAM	UNI	stainnorm	N/A	N/A	N/A	N/A	N/A	0.41±0.29 [0.16,0.66]	0.54±0.26 [0.31,0.77]	**1.00±0.00 [1.00,1.00]**	0.41±0.29 [0.16,0.66]
Patch	CONCH	default	0.06±0.03 [0.04,0.08]	0.37±0.07 [0.30,0.43]	64	0.5	(128, 64)	0.38±0.10 [0.29,0.46]	0.54±0.11 [0.45,0.63]	**1.00±0.00 [1.00,1.00]**	0.38±0.10 [0.29,0.46]
Patch	ResNet50	default	0.02±0.01 [0.01,0.02]	0.40±0.18 [0.24,0.56]	64	0.3	(64, 32)	0.47±0.47 [0.06,0.88]	0.53±0.44 [0.15,0.91]	0.80±0.45 [0.41,1.19]	0.47±0.47 [0.06,0.88]
Patch	ResNet50	gray	0.03±0.01 [0.02,0.04]	0.40±0.13 [0.28,0.51]	128	0.3	(64, 32)	0.40±0.25 [0.18,0.62]	0.53±0.31 [0.25,0.80]	0.80±0.45 [0.41,1.19]	0.40±0.25 [0.18,0.62]
Patch	UNI	gray	0.06±0.04 [0.02,0.09]	0.40±0.08 [0.33,0.47]	128	0.3	(32, 16)	0.40±0.26 [0.17,0.63]	0.53±0.26 [0.30,0.76]	**1.00±0.00 [1.00,1.00]**	0.40±0.26 [0.17,0.63]
Patch	CONCH	gray	0.04±0.02 [0.02,0.06]	0.41±0.03 [0.39,0.44]	128	0.5	(32, 16)	0.36±0.08 [0.30,0.43]	0.53±0.08 [0.46,0.60]	**1.00±0.00 [1.00,1.00]**	0.36±0.08 [0.30,0.43]
Patch	CONCH	stainnorm	0.10±0.06 [0.05,0.15]	0.43±0.05 [0.38,0.47]	128	0.3	(32, 16)	0.36±0.17 [0.22,0.51]	0.52±0.18 [0.36,0.68]	**1.00±0.00 [1.00,1.00]**	0.36±0.17 [0.22,0.51]
mCLAM	UNI	stainnorm	N/A	N/A	N/A	N/A	N/A	0.36±0.13 [0.25,0.48]	0.52±0.14 [0.40,0.65]	**1.00±0.00 [1.00,1.00]**	0.36±0.13 [0.25,0.48]
Patch	CONCH	gray	0.10±0.06 [0.05,0.15]	0.42±0.05 [0.38,0.47]	128	0.3	(32, 16)	0.35±0.08 [0.28,0.43]	0.52±0.09 [0.43,0.60]	**1.00±0.00 [1.00,1.00]**	0.35±0.08 [0.28,0.43]
CLAM	ResNet50	gray	N/A	N/A	N/A	N/A	N/A	0.38±0.27 [0.14,0.61]	0.51±0.24 [0.30,0.72]	**1.00±0.00 [1.00,1.00]**	0.38±0.27 [0.14,0.61]
Patch	CONCH	gray	0.11±0.11 [0.01,0.20]	0.42±0.07 [0.36,0.49]	128	0.5	(128, 64)	0.35±0.16 [0.21,0.49]	0.51±0.17 [0.35,0.66]	**1.00±0.00 [1.00,1.00]**	0.35±0.16 [0.21,0.49]
Patch	ResNet50	gray	0.03±0.04 [-0.00,0.06]	0.31±0.04 [0.27,0.35]	64	0.5	(32, 16)	0.41±0.36 [0.09,0.73]	0.50±0.39 [0.16,0.85]	0.80±0.45 [0.41,1.19]	0.41±0.36 [0.09,0.73]
CLAM	ResNet50	default	N/A	N/A	N/A	N/A	N/A	0.39±0.32 [0.11,0.66]	0.50±0.31 [0.23,0.78]	**1.00±0.00 [1.00,1.00]**	0.39±0.32 [0.11,0.66]
Patch	UNI	default	0.03±0.02 [0.01,0.05]	0.34±0.11 [0.25,0.44]	128	0.3	(64, 32)	0.41±0.41 [0.05,0.77]	0.49±0.41 [0.13,0.85]	0.80±0.45 [0.41,1.19]	0.41±0.41 [0.05,0.77]
Patch	ResNet50	default	0.02±0.01 [0.00,0.03]	0.40±0.16 [0.27,0.54]	128	0.3	(64, 32)	0.40±0.40 [0.05,0.75]	0.48±0.40 [0.12,0.83]	0.80±0.45 [0.41,1.19]	0.40±0.40 [0.05,0.75]
Patch	ResNet50	gray	0.14±0.26 [-0.09,0.36]	0.45±0.13 [0.33,0.56]	64	0.5	(64, 32)	0.35±0.25 [0.13,0.57]	0.48±0.28 [0.23,0.73]	**1.00±0.00 [1.00,1.00]**	0.35±0.25 [0.13,0.57]
CLAM	CONCH	default	N/A	N/A	N/A	N/A	N/A	0.35±0.27 [0.11,0.59]	0.47±0.33 [0.18,0.76]	0.80±0.45 [0.41,1.19]	0.35±0.27 [0.11,0.59]

Patch	ResNet50	default	0.01±0.01 [0.01,0.02]	0.43±0.19 [0.26,0.60]	64	0.3	(128, 64)	0.39±0.42 [0.02,0.76]	0.46±0.42 [0.09,0.83]	0.80±0.45 [0.41,1.19]	0.39±0.42 [0.02,0.76]
Patch	UNI	default	0.02±0.02 [0.00,0.04]	0.35±0.13 [0.24,0.46]	64	0.5	(128, 64)	0.36±0.38 [0.03,0.70]	0.46±0.36 [0.14,0.77]	0.80±0.45 [0.41,1.19]	0.36±0.38 [0.03,0.70]
mMIL	UNI	stainnorm	N/A	N/A	N/A	N/A	N/A	0.30±0.07 [0.24,0.36]	0.46±0.08 [0.38,0.53]	**1.00±0.00 [1.00,1.00]**	0.30±0.07 [0.24,0.36]
Patch	UNI	default	0.02±0.02 [0.00,0.04]	0.33±0.08 [0.27,0.40]	128	0.5	(64, 32)	0.38±0.39 [0.03,0.72]	0.45±0.44 [0.07,0.83]	0.60±0.55 [0.12,1.08]	0.38±0.39 [0.03,0.72]
Patch	ResNet50	default	0.03±0.05 [-0.01,0.08]	0.34±0.15 [0.21,0.47]	128	0.5	(32, 16)	0.36±0.41 [0.01,0.72]	0.44±0.42 [0.07,0.80]	0.80±0.45 [0.41,1.19]	0.36±0.41 [0.01,0.72]
Patch	ResNet50	default	0.01±0.01 [0.00,0.02]	0.39±0.17 [0.24,0.54]	128	0.5	(128, 64)	0.35±0.39 [0.01,0.69]	0.43±0.41 [0.06,0.79]	0.80±0.45 [0.41,1.19]	0.35±0.39 [0.01,0.69]
Patch	ResNet50	gray	0.13±0.24 [-0.08,0.34]	0.42±0.12 [0.31,0.53]	64	0.3	(32, 16)	0.35±0.39 [0.01,0.69]	0.43±0.40 [0.08,0.78]	**1.00±0.00 [1.00,1.00]**	0.35±0.39 [0.01,0.69]
Patch	UNI	default	0.04±0.03 [0.02,0.06]	0.33±0.09 [0.25,0.41]	128	0.5	(128, 64)	0.34±0.37 [0.01,0.67]	0.42±0.39 [0.09,0.76]	0.80±0.45 [0.41,1.19]	0.34±0.37 [0.01,0.67]
Patch	UNI	default	0.04±0.03 [0.01,0.07]	0.39±0.17 [0.24,0.54]	64	0.3	(64, 32)	0.34±0.41 [-0.02,0.70]	0.41±0.42 [0.04,0.78]	0.60±0.55 [0.12,1.08]	0.34±0.41 [-0.02,0.70]
Patch	ResNet50	default	0.02±0.02 [0.00,0.03]	0.38±0.18 [0.22,0.54]	128	0.5	(64, 32)	0.34±0.41 [-0.02,0.70]	0.41±0.40 [0.06,0.77]	0.80±0.45 [0.41,1.19]	0.34±0.41 [-0.02,0.70]
Patch	ResNet50	default	0.02±0.03 [0.00,0.05]	0.36±0.16 [0.21,0.50]	128	0.3	(128, 64)	0.33±0.37 [0.00,0.66]	0.41±0.38 [0.08,0.74]	0.80±0.45 [0.41,1.19]	0.33±0.37 [0.00,0.66]
Patch	ResNet50	default	0.01±0.01 [0.01,0.02]	0.37±0.11 [0.27,0.47]	128	0.3	(32, 16)	0.33±0.37 [0.00,0.66]	0.41±0.38 [0.08,0.74]	0.80±0.45 [0.41,1.19]	0.33±0.37 [0.00,0.66]
Patch	ResNet50	default	0.02±0.02 [0.01,0.04]	0.40±0.19 [0.23,0.56]	64	0.5	(64, 32)	0.33±0.39 [-0.01,0.67]	0.40±0.41 [0.04,0.76]	0.80±0.45 [0.41,1.19]	0.33±0.39 [-0.01,0.67]
mMIL	UNI	gray	N/A	N/A	N/A	N/A	N/A	0.25±0.09 [0.17,0.33]	0.39±0.13 [0.28,0.50]	**1.00±0.00 [1.00,1.00]**	0.25±0.09 [0.17,0.33]
Patch	UNI	default	0.06±0.04 [0.02,0.09]	0.31±0.09 [0.23,0.39]	128	0.5	(32, 16)	0.31±0.38 [-0.03,0.64]	0.38±0.38 [0.04,0.72]	0.80±0.45 [0.41,1.19]	0.31±0.38 [-0.03,0.64]
Patch	UNI	default	0.03±0.03 [0.01,0.05]	0.35±0.09 [0.27,0.42]	64	0.5	(64, 32)	0.29±0.38 [-0.04,0.62]	0.37±0.37 [0.04,0.70]	0.80±0.45 [0.41,1.19]	0.29±0.38 [-0.04,0.62]
Patch	ResNet50	default	0.01±0.01 [0.00,0.02]	0.33±0.14 [0.21,0.45]	64	0.5	(32, 16)	0.32±0.45 [-0.07,0.71]	0.35±0.49 [-0.07,0.78]	0.40±0.55 [-0.08,0.88]	0.32±0.45 [-0.07,0.71]
Patch	ResNet50	default	0.02±0.02 [0.00,0.04]	0.36±0.13 [0.25,0.48]	64	0.3	(32, 16)	0.31±0.42 [-0.06,0.67]	0.35±0.45 [-0.04,0.75]	0.60±0.55 [0.12,1.08]	0.31±0.42 [-0.06,0.67]
Patch	ResNet50	gray	0.12±0.23 [-0.08,0.32]	0.42±0.15 [0.29,0.55]	128	0.5	(64, 32)	0.26±0.32 [-0.02,0.54]	0.34±0.38 [0.00,0.67]	0.60±0.55 [0.12,1.08]	0.26±0.32 [-0.02,0.54]
CLAM	UNI	gray	N/A	N/A	N/A	N/A	N/A	0.22±0.17 [0.07,0.38]	0.34±0.23 [0.14,0.54]	**1.00±0.00 [1.00,1.00]**	0.22±0.17 [0.07,0.38]
Patch	UNI	default	0.03±0.03 [0.00,0.05]	0.31±0.09 [0.23,0.38]	64	0.5	(32, 16)	0.25±0.34 [-0.05,0.54]	0.32±0.37 [-0.01,0.64]	0.60±0.55 [0.12,1.08]	0.25±0.34 [-0.05,0.54]
Patch	ResNet50	default	0.03±0.02 [0.01,0.04]	0.38±0.13 [0.26,0.49]	64	0.5	(128, 64)	0.25±0.33 [-0.05,0.54]	0.32±0.35 [0.01,0.63]	0.80±0.45 [0.41,1.19]	0.25±0.33 [-0.05,0.54]
Patch	ResNet50	gray	0.09±0.07 [0.03,0.16]	0.38±0.12 [0.27,0.49]	128	0.3	(32, 16)	0.21±0.18 [0.05,0.37]	0.32±0.24 [0.11,0.53]	0.80±0.45 [0.41,1.19]	0.21±0.18 [0.05,0.37]

mCLAM	UNI	gray	N/A	N/A	N/A	N/A	N/A	0.18±0.06 [0.12,0.23]	0.30±0.09 [0.22,0.37]	**1.00±0.00 [1.00,1.00]**	0.18±0.06 [0.12,0.23]
Patch	UNI	default	0.01±0.02 [0.00,0.03]	0.35±0.14 [0.23,0.47]	128	0.3	(128, 64)	0.25±0.43 [-0.13,0.63]	0.28±0.44 [-0.11,0.66]	0.40±0.55 [-0.08,0.88]	0.25±0.43 [-0.13,0.63]
Patch	UNI	default	0.01±0.02 [0.00,0.03]	0.38±0.17 [0.22,0.53]	64	0.3	(128, 64)	0.24±0.43 [-0.15,0.62]	0.26±0.43 [-0.12,0.64]	0.40±0.55 [-0.08,0.88]	0.24±0.43 [-0.15,0.62]
mMIL	ResNet50	gray	N/A	N/A	N/A	N/A	N/A	0.15±0.06 [0.09,0.21]	0.26±0.09 [0.18,0.34]	**1.00±0.00 [1.00,1.00]**	0.15±0.06 [0.09,0.21]
mCLAM	UNI	default	N/A	N/A	N/A	N/A	N/A	0.18±0.23 [-0.03,0.38]	0.25±0.31 [-0.02,0.52]	0.60±0.55 [0.12,1.08]	0.18±0.23 [-0.03,0.38]
mMIL	ResNet50	default	N/A	N/A	N/A	N/A	N/A	0.15±0.11 [0.05,0.25]	0.25±0.15 [0.11,0.38]	**1.00±0.00 [1.00,1.00]**	0.15±0.11 [0.05,0.25]
mMIL	ResNet50	stainnorm	N/A	N/A	N/A	N/A	N/A	0.14±0.03 [0.11,0.17]	0.25±0.05 [0.21,0.29]	**1.00±0.00 [1.00,1.00]**	0.14±0.03 [0.11,0.17]
Patch	UNI	default	0.02±0.02 [0.00,0.04]	0.31±0.10 [0.22,0.39]	64	0.3	(32, 16)	0.18±0.33 [-0.12,0.47]	0.22±0.38 [-0.11,0.54]	0.40±0.55 [-0.08,0.88]	0.18±0.33 [-0.12,0.47]
mMIL	CONCH	default	N/A	N/A	N/A	N/A	N/A	0.13±0.07 [0.07,0.18]	0.22±0.10 [0.13,0.31]	**1.00±0.00 [1.00,1.00]**	0.13±0.07 [0.07,0.18]
mMIL	UNI	default	N/A	N/A	N/A	N/A	N/A	0.11±0.04 [0.08,0.15]	0.20±0.06 [0.15,0.25]	**1.00±0.00 [1.00,1.00]**	0.11±0.04 [0.08,0.15]
mCLAM	CONCH	default	N/A	N/A	N/A	N/A	N/A	0.11±0.09 [0.03,0.18]	0.18±0.14 [0.06,0.31]	0.80±0.45 [0.41,1.19]	0.11±0.09 [0.03,0.18]
Patch	UNI	default	0.05±0.06 [0.00,0.10]	0.34±0.10 [0.25,0.43]	128	0.3	(32, 16)	0.09±0.18 [-0.06,0.25]	0.14±0.25 [-0.08,0.36]	0.40±0.55 [-0.08,0.88]	0.09±0.18 [-0.06,0.25]
CLAM	UNI	default	N/A	N/A	N/A	N/A	N/A	0.00±0.00 [0.00,0.00]	0.00±0.00 [0.00,0.00]	0.00±0.00 [0.00,0.00]	0.00±0.00 [0.00,0.00]
